# Strain-switchable field-induced superconductivity

**DOI:** 10.1126/sciadv.adj5200

**Published:** 2023-11-24

**Authors:** Joshua J. Sanchez, Gilberto Fabbris, Yongseong Choi, Jonathan M. DeStefano, Elliott Rosenberg, Yue Shi, Paul Malinowski, Yina Huang, Igor I. Mazin, Jong-Woo Kim, Jiun-Haw Chu, Philip J. Ryan

**Affiliations:** ^1^ Department of Physics, Massachusetts Institute of Technology, Cambridge, MA 02139, USA.; ^2^ Department of Physics, University of Washington, Seattle, WA 98195, USA.; ^3^ Advanced Photon Source, Argonne National Laboratory, Lemont, IL 60439, USA.; ^4^ Department of Physics, Cornell University, Ithaca, NY 14853, USA.; ^5^ Department of Physics, Zhejiang University of Science and Technology, Hangzhou 310023, People’s Republic of China.; ^6^ Department of Physics and Astronomy and Quantum Science and Engineering Center, George Mason University, Fairfax, VA 22030, USA.

## Abstract

Field-induced superconductivity is a rare phenomenon where an applied magnetic field enhances or induces superconductivity. Here, we use applied stress as a control switch between a field-tunable superconducting state and a robust non–field-tunable state. This marks the first demonstration of a strain-tunable superconducting spin valve with infinite magnetoresistance. We combine tunable uniaxial stress and applied magnetic field on the ferromagnetic superconductor Eu(Fe_0.88_Co_0.12_)_2_As_2_ to shift the field-induced zero-resistance temperature between 4 K and a record-high value of 10 K. We use x-ray diffraction and spectroscopy measurements under stress and field to reveal that strain tuning of the nematic order and field tuning of the ferromagnetism act as independent control parameters of the superconductivity. Combining comprehensive measurements with DFT calculations, we propose that field-induced superconductivity arises from a novel mechanism, namely, the uniquely dominant effect of the Eu dipolar field when the exchange field splitting is nearly zero.

## Teaser

Strain-tunable field-induced superconductivity is demonstrated at an ultra-high temperature in a ferromagnetic superconductor.

## INTRODUCTION

Switching between distinct electronic phases in quantum materials by external tuning parameters is a central focus of condensed matter physics, both to study how competing orders interact and to drive technological development (*1*). One rich research area is tuning systems with both ferromagnetism and superconductivity. The interaction of these antagonistic phases leads to unusual phenomena, such as spontaneous magnetic vortices (*2*–*4*) and spin-polarized supercurrents (*5*–*7*), which hold promise for superconducting spintronics technologies and energy-efficient data storage. Much attention has focused on superconducting spin valves, i.e., heterostructures with ferromagnetic layers surrounding a superconducting layer (*5*–*8*). An applied magnetic field switches the sandwiching ferromagnetic layers between parallel and antiparallel alignment, which strongly tunes the magnetic pairbreaking effect and effectively turns the superconductivity on and off. This enables the ultimate switchability of magneto-transport, between a resistive and zero-resistance state, thus achieving infinite magnetoresistance and the possibility of low-energy dissipation information technologies (*5*).

The development of these technologies is impeded by the very low temperatures required to implement them. Besides artificial heterostructures, a handful of single-crystal materials exhibit field-induced superconductivity, including several Eu- and U-based superconductors (*9*–*14*) and organic superconductors (*15*, *16*). In these systems as well as in thin-film superconducting spin valves, the zero-resistance temperature *T*_0_ is often below 1 K, limiting their practical application. The current highest field-induced superconductivity temperature is in the chemically doped Eu-based iron pnictide superconductor, EuFe_2_As_2_. Like other iron pnictide superconductors, EuFe_2_As_2_ exhibits an electronic nematic transition, which creates orthorhombic structural twin domains. The suppression of nematicity by chemical doping results in the emergence of both superconductivity, with an onset temperature *T*_SC_ reaching 18 to 30 K at optimal doping (*17*–*22*), and Eu ferromagnetism, with *T*_FM_ = 16 to 20 K (*2*, *23*–*26*). The similar ordering temperatures of the two antagonistic phases implies a potentially strong competition between them. For Co- and Rh-doped samples, a large reentrant resistivity appears below *T*_FM_ as the Eu magnetic flux disrupts the nascent superconductivity, pushing *T*_0_ far below *T*_SC_. Unexpectedly, applying a small in-plane magnetic field (μ_0_*H* < 0.5 T) to these materials raises *T*_0_ from ~5 K to ~6 to 7 K (*27*, *28*). To date, the underlying mechanism of this field-induced superconductivity has not been determined, nor has the effect been optimized to enhance *T*_0_ to its limit.

**Fig. 1.**
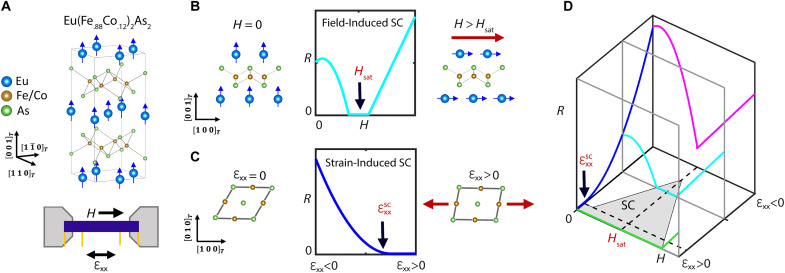
Strain and field-tunable ferromagnetic superconductivity. (**A**) Eu(Fe_.88_Co_.12_)_2_As_2_ consists of stacked planes of Eu and doped FeAs layers, with the former exhibiting ferromagnetism (FM; *T*_FM_ = 17 K) and the latter hosting both nematicity (N; *T*_S_ = 68 K) and superconductivity (SC; *T*_SC_ = 19 K). Below *T*_FM_, the coexistence and competition between the three phases enables exceptional tunability of the superconductivity. (**B**) A small in-plane magnetic field reorients the Eu moments from out of plane to in plane, reducing the magnetic flux through the FeAs layers. A zero-resistance (*R* = 0) state occurs in the vicinity of the full saturation of the moments in plane (at *H* = *H*_sat_), demonstrating field-induced superconductivity. (**C**) As in other iron-pnictide superconductors, the N/SC phase competition enables an effective strain tuning of superconductivity via strain tuning the lattice-coupled nematic order. Tensile strain (ɛ_xx_ > 0) along the FeAs bonding direction suppresses the nematicity-driven orthorhombicity along the FeFe bonding direction. This enhances superconductivity, with the entrance into the *R* = 0 state labeled as $\varepsilon_{\mathrm{xx}}^{\mathrm{sc}}$. (**D**) Combined strain and field tuning of the resistivity defines an *R* = 0 superconducting region of the phase diagram (gray) at one fixed temperature, with a precise shape that depends on the (temperature-dependent) values of $\varepsilon_{\mathrm{xx}}^{\mathrm{sc}}$ and *H*_sat_. For fields from *H* = 0 to *H* = *H*_sat_, strain selects between an always metallic state (magenta), an always superconducting state (green), and a field-induced superconducting state (cyan). Thus, strain acts like a toggle switch for the phase field tunability.

Here, we demonstrate field-induced superconductivity in 12% Co-doped EuFe_2_As_2_ at *T*_0_ = 9 K, which can be enhanced up to at least 10 K or suppressed to 4 K using in situ applied uniaxial stress. To our knowledge, this is the highest reported temperature of magnetic field–induced superconductivity in any material. Doped EuFe_2_As_2_ exists as a natural-grown atomic limit of the thin-film superconducting spin valve architecture, with alternating ferromagnetic Eu and superconducting/nematic FeAs layers (Fig. 1A). We combine synchrotron x-ray techniques with transport measurements to reveal that strain tuning nematicity and field tuning the Eu moments act as independent superconductivity control parameters (Fig. 1, B and C). This strain tunability, combined with the high temperature and low switching field, makes doped EuFe_2_As_2_ an exciting platform for potential superconducting spintronics applications, and we propose a new superconducting switch device concept using this strain tunability.

Finally, we perform density functional theory (DFT) calculations to show that the cancellation of Eu-Fe ferromagnetic and antiferromagnetic exchange interactions results in a very weak exchange field, which solves the mystery of how the superconducting order can coexist with ferromagnetism. In the near absence of the exchange field, the Eu dipole field has a dominant effect on the superconductivity. We introduce a new mechanism for field-induced superconductivity, whereby an external field reorients this internal dipole field from the direction of lower to higher upper critical field *H*_c2_, enabling the applied field to enhance *T*_0_. We consider how this novel mechanism could be realized in other systems, including in two-dimensional (2D) systems and at even higher temperatures.

## RESULTS

### Strain-switchable field-induced superconductivity

Single-crystal samples of 12% Co-doped EuFe_2_As_2_ were grown using Sn flux (see Materials and Methods). Using a (Fe,Co)–rich, nonstoichiometric growth composition yielded samples with increased superconducting transition temperatures relative to stoichiometric-grown samples (Materials and Methods and fig. S1) (*27*). Samples 1 and 2 were selected from different growth batches and were prepared identically as matchsticks to measure the inline resistivity ρ_xx_ (Fig. 1A, bottom). To better compare the field and strain tuning of the resistivity, transport data are normalized to the zero-field freestanding resistivity at *T* = 25 K, with ρ/ρ_0_ = ρ_xx_(*T*, μ_0_*H*, ɛ_xx_)/ρ_xx_(25 K,0,0).

**Fig. 2.**
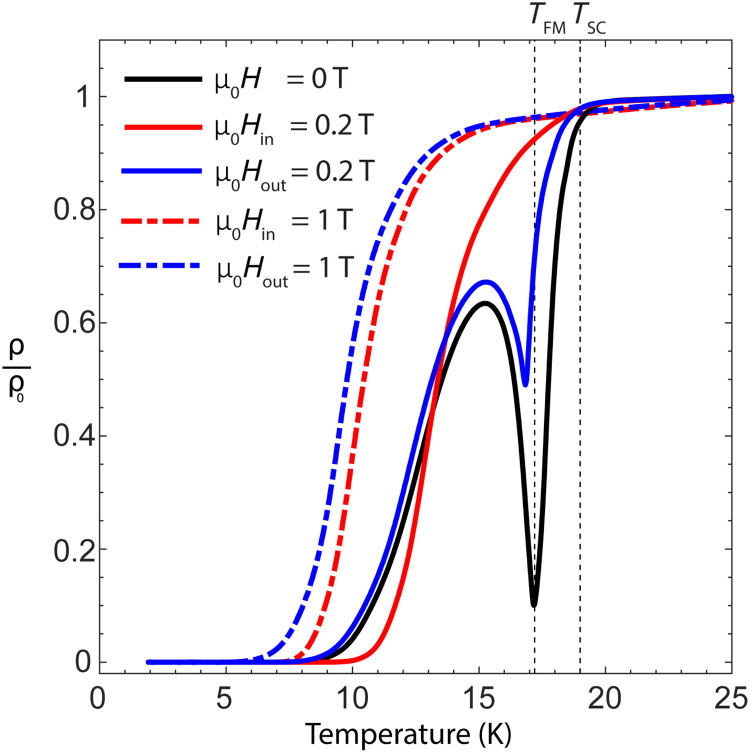
Zero-strain field-induced superconductivity. Sample 1 resistivity versus temperature for zero applied field (black) and μ_0_*H* = 0.2 T (solid line) and 1 T (dashed line) applied in plane (red) and out of plane (blue). For μ_0_*H* = 0.2 T applied in plane, the zero-resistivity temperature rises from *T*_0_ = 7.5 to 9.0 K.

In the freestanding state, sample 1 was cooled through the superconducting (*T*_SC_ = 19 K, onset temperature at ρ/ρ_0_ = 0.99) and ferromagnetic (*T*_FM_ = 17.2 K, at the minimum of ρ/ρ_0_) transitions under zero field (Fig. 2, black), reaching ρ/ρ_0_= 0 at *T*_0_ = 7.5 K. Temperature sweeps were repeated with fixed magnetic field applied either in plane (Fig. 2, red) or out of plane (Fig. 2, blue). The latter is found to increase the resistivity and reduce the value of *T*_0_. In sharp contrast, an in-plane field is far more detrimental to superconductivity between *T*_SC_ and *T*_FM_, but zero resistance is reached at an enhanced value of *T*_0_ = 9.0 K for μ_0_*H* = 0.2 T, thus demonstrating field-induced superconductivity.

**Fig. 3.**
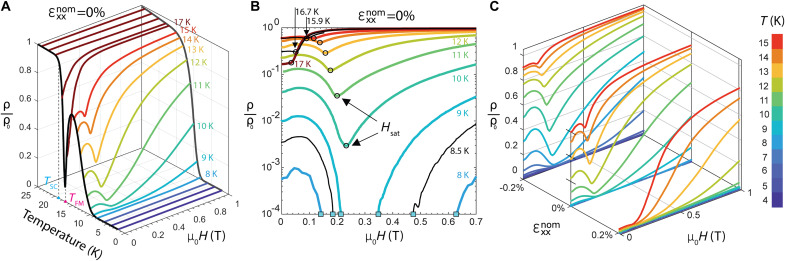
Strain-tunable field-induced superconductivity. (**A**) Freestanding (ɛ_xx_ = 0) resistivity versus temperature at fixed in-plane applied field (μ_0_*H* = 0 T, black; μ_0_*H* = 1 T, gray) and resistivity versus in-plane field at fixed temperature. Onset of superconducting transition (*T*_SC_ = 19 K) and ferromagnetic order (*T*_FM_ = 17.2 K) indicated. (**B**) Same resistivity versus field data as in (A) plotted against logarithm *y* axis, with additional data at three non-integer temperatures (black). Cyan markers indicate entrance and exit from zero-resistance state for *T* = 8 to 9 K. Minimum of resistivity for *T* = 10 to 16.7 K and inflection point at 17 K marked by black circles, corresponding to the in-plane saturation field *H*_sat_ needed to align the Eu moments in plane. Entrance and exit from zero-resistance state marked by cyan squares. (**C**) Resistivity versus field at fixed temperatures (4 to 15 K) for one tensile and one compressive strain state and corresponding freestanding values from (A). Field range of zero resistance shown in Fig. 4 (shaded).

**Fig. 4.**
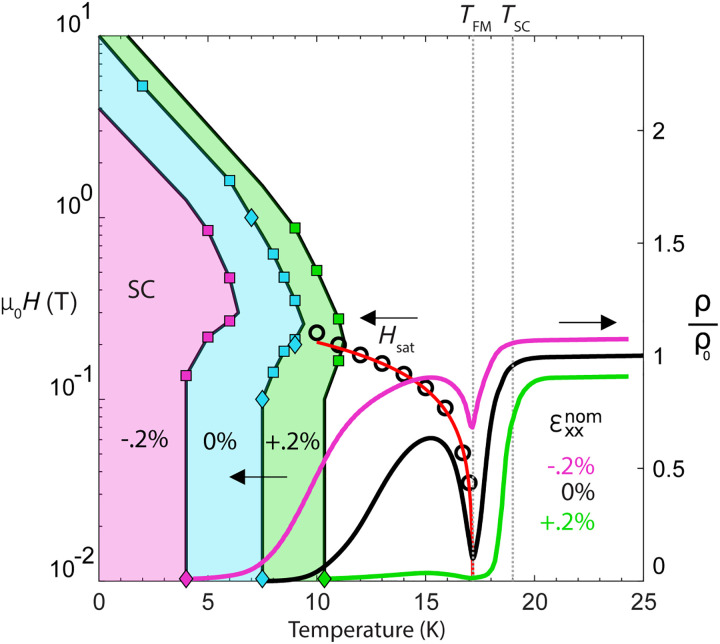
Strain and field-tunable phase diagram. (**Right**) Resistivity versus temperature for the zero-strain state (same as black curve in Figs. 2 and 3) and for the tensile (green) and compressive (magenta) strain states in Fig. 3C. (**Left**) Phase boundary between ρ > 0 and ρ = 0 states under zero strain (cyan), tension (green), and compression (magenta), determined by resistivity versus temperature data (diamonds) and resistivity versus magnetic field (squares) from Fig. 3 and fig. S5. Field-induced superconductivity indicated by shaded areas for each strain state. Eu in-plane saturation field *H*_sat_ taken from minimum of magnetoresistance in Fig. 3B versus temperature (black circles), with mean-field fit line (red).

Figure 3A shows ρ/ρ_0_ versus temperature at fixed in-plane field (μ_0_*H* = 0 T and μ_0_*H* = 1 T) and ρ/ρ_0_ versus field at fixed temperature for sample 1. For *T* > *T*_FM_, an applied field up to 1 T acts only to increase the resistivity. For *T* < *T*_0_, ρ/ρ_0_ = 0 up to 1 T. However, for *T*_FM_ > *T* > *T*_0_, the minimum resistivity value is reached at finite field. As we will show, this resistivity minimum corresponds to the full in-plane saturation of the Eu moments, and we mark this field value as *H*_sat_ (Fig. 3B, black circles). Figure 4 presents *H*_sat_ versus temperature, which follows a square root temperature dependence, $H_{\mathrm{sat}}\propto \sqrt{T_{\mathrm{FM}}-T}$, indicating the mean-field behavior of the Eu magnetic ordering. For 9 K > *T* > 7.5 K, zero resistance can be induced in the vicinity of *H*_sat_.

Following these measurements, sample 1 was mounted to a uniaxial stress device (see Materials and Methods and figs. S6 and S7). Stress was applied along the Fe-As bonding direction, inducing strain in both B_1g_ and A_1g_ symmetry channels. The sample was initially cooled under zero device voltage to base temperature and then was slowly warmed under large fixed tension or compression to yield the resistivity versus temperature curves in Fig. 4 (right). We find that *T*_SC_ varies monotonically with strain and is tuned by ~1 K, revealing the tunability of the nematicity/superconductivity phase competition in line with previous work in BaFe_2_As_2_ (*29*, *30*). Below *T*_FM_, the resistivity is especially tunable, and *T*_0_ is tuned by ~3 K (and fully suppressed under maximum compression; see fig. S6). However, the Eu magnetic order is apparently independent of strain, as *T*_FM_ is constant across this strain range.

Applying field at fixed temperature and stress (Fig. 3C) enables the construction of a superconductivity strain field-tunable phase diagram (Fig. 4). Field-induced superconductivity is accessible in a temperature window from 7.5 to 9 K under zero strain, with a maximum near 11 K under tension and a minimum of 4 K under compression. With decreasing temperature, an increasing Eu magnetic moment causes the ferromagnetism to have a larger influence on the superconductivity, and so the zero-resistance phase volume increases under compression and decreases under tension. Across this range, the onset field only varies between μ_0_*H* = 0.1 T and 0.3 T; we note that this is a substantial qualitative difference from UTe_2_ where pressure tuning can shift the critical field by many tesla (*14*).

### Strain and magnetic field: Independent tuning knobs of superconductivity

To further identify the independence of strain and magnetic field for tuning superconductivity, as well as to resolve the mechanism of the field-induced superconductivity, we performed transport measurements under applied strain concurrent with either x-ray diffraction (XRD) or x-ray magnetic circular dichroism (XMCD) at the Advanced Photon Source. XMCD is a powerful tool to study ferromagnetic superconductors. It provides element-specific magnetic information, and, carried out in fluorescence mode, any diamagnetic shielding from the superconductivity is avoided.

**Fig. 5.**
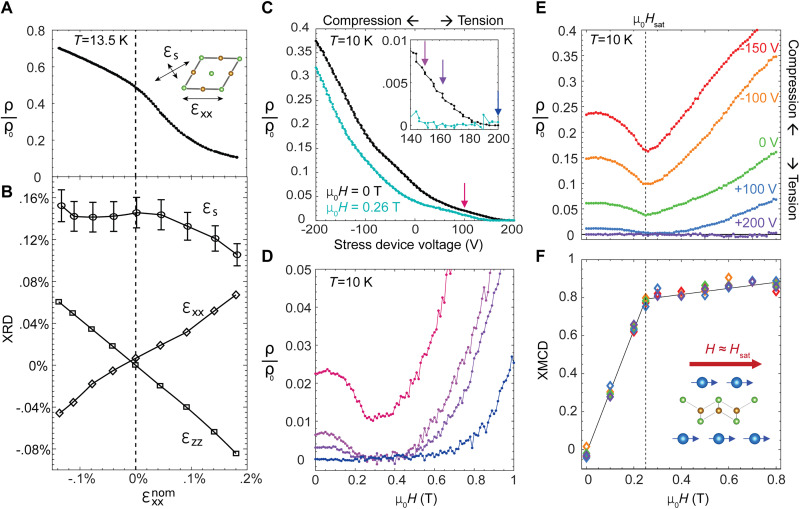
X-ray characterization of independent strain and field tuning. (**A** and **B**) Fixed temperature (*T* = 13.5 K) strain sweep (compressive to tensile) with simultaneous (A) resistivity measurements and (B) XRD measurements of the inline strain ɛ_xx_, the out-of-plane strain ɛ_zz_, and the nematicity-driven spontaneous orthorhombicity ɛ_S_ (see Materials and Methods for definitions). (**C**) Resistivity versus strain device voltage at *T* = 10 K under in-plane applied field of μ_0_*H* = 0 T and μ_0_*H* = 0.26 T. Inset shows high tension range. The voltage range in (C) corresponds approximately to the range of $\varepsilon_{\mathrm{xx}}^{\mathrm{nom}}$ in (A) and (B), but could not be simultaneously measured due to sample chamber restrictions. (**D**) Resistivity versus applied in-plane field at fixed strain values corresponding to colored arrows in (C) (inset). (**E** and **F**) The simultaneously collected resistivity (E) and XMCD (F) versus applied field at *T* = 10 K for five fixed strain values (see Materials and Methods for XMCD normalization details). Eu moment saturation coincides with minimum of resistivity at *H* = *H*_sat_. Voltages listed in (E) and (F) correspond to slightly greater tension states than corresponding voltages in (C) due to different thermal hysteresis in the piezo actuators between the two measurements. A small negative background magnetoresistance was removed from data in (E) (see Materials and Methods). Error bars in (B) on ɛ_S_ represent error propagation of Gaussian fits to the split [1 1 8]_T_ reflection peak (see Materials and Methods), while error bars on ɛ_xx_ and ɛ_zz_ are smaller than marker size.

We performed XRD measurements on sample 2 at *T* = 13.5 K, just below the maximum of the reentrant resistivity, across a range of strain. The linearity of the inline strain ɛ_xx_ confirms a constant strain transmission (Fig. 5B). We also measured the B_2g_-symmetry spontaneous orthorhombicity ɛ_S_, which is a proxy of the nematic order (see Materials and Methods and fig. S2) (*31*). Under applied tension, the magnitude of ɛ_S_ is suppressed by up to 30%, coinciding with a dramatic decrease in the resistivity (Fig. 5A). Under compression, ɛ_S_ is roughly constant as the resistivity increases, suggesting that the saturated nematicity suppresses the superconductivity. This strain dependence of nematicity is consistent with the combination effect of the induced A_1g_ and B_1g_ strains, where the latter acts as a transverse field that suppresses nematicity quadratically. Thus, we effectively strain-tune superconductivity via its competition with the strain-tunable nematicity and the associated antiferromagnetic order (*30*, *32*).

Field-induced superconductivity was observed at *T* = 10 K under both fixed-strain and fixed-field conditions. With zero field, the resistivity can be strain-tuned from ρ/ρ_0_ = 5% under zero strain, to ρ/ρ_0_ = 40% at maximum compression, and ρ/ρ_0_ = 0% with maximum tension (Fig. 5C). Thus, tensile strain can effectively raise the superconducting transition to at least 10 K. The application of an in-plane magnetic field (μ_0_*H* = 0.26 T) decreases the resistivity at all strain states, and zero resistivity is obtained at roughly 75% of the maximum applied tension. Thus, tensile strain and magnetic field can work together to raise the transition temperature even higher. Figure 5D shows resistivity versus applied magnetic field at four fixed tension values, where a narrow strain range permits field-induced superconductivity.

To investigate the origin of the field-induced superconductivity, we performed simultaneous resistivity and XMCD measurements versus field at five fixed strain states between maximum compression and tension (Fig. 5, E and F). Here, the XMCD signal is proportional to the Eu magnetization along the field direction (see Materials and Methods). As the Eu ferromagnetic moments are spontaneously ordered along the *c* axis, the Eu in-plane moment (and XMCD signal) is initially nearly zero under zero field. For all strains, increasing the magnetic field linearly increases the in-plane moment toward saturation at μ_0_*H*_sat_ = 0.25 T, coinciding with the magnetoresistance minimum. From this, we conclude that the Eu moment reorientation toward the in-plane direction is intimately connected to field-induced superconductivity. Despite the large change in the zero-field resistivity with strain, there is no apparent strain-induced change in either the saturation field value or saturation XMCD value. This strain independence is unexpected given that the localized Eu 4f electrons presumably order with assistance from the strain-sensitive Fe 3d electrons via an RKKY interaction (*33*). As strain does not affect the Eu magnetic order, and as strain is far more effective than magnetic field in tuning the nematic order in this material system (*34*), we find that strain and field act as independent tuning parameters of superconductivity.

### Dipole coupling and the mechanism of field-induced superconductivity

The antiferromagnetic EuFe_2_As_2_ parent compound has a strong biquadratic interaction between Eu and Fe moments, manifesting as a large magnetostructural coupling (*34*–*36*). The presumed weakening of this coupling with doping causes the Eu moments to become spontaneously polarized along the *c* axis, which would naively be expected to create a strong exchange splitting that destroys the superconductivity. The Jaccarino-Peter effect (*37*) has often been invoked to explain field-induced superconductivity in s-wave superconductors, including in Eu-based Chevrel phases (*9*, *10*) and organic superconductors (*15*, *16*, *38*). Here, the Zeeman splitting induced by an external field compensates the internal exchange-bias splitting, resulting in superconductivity. However, in our experiment, the exchange-bias field is parallel to the external field, so a Jaccarino-Peter compensation is not possible. Instead, two other mechanisms contribute to the exchange splitting induced in the Fe bands: the Hund’s rule coupling of Eu f- and d-orbitals, with the latter overlapping with Fe d-orbitals and inducing a polarization parallel to Eu f moments, and the Schrieffer-Wolfe coupling of Eu f- and Fe d-orbitals, which leads to an antiparallel polarization. To characterize these two effects, we performed DFT calculations using the Wien2K package (*39*, *40*) for Eu moments fully polarized in plane (figs. S8 to S10). We find that both show high sensitivity to the Hubbard U on Eu sites, and as these two interactions have opposite signs, the induced splitting of Fe bands is relatively small and varying in sign and amplitude over the Fermi surface. This “accidental cancelation” gives a reasonable explanation for the coexistence of superconductivity and ferromagnetism. Above *T*_FM_, this cancellation is lifted as the Eu moments disorder, which also explains the flipped field preference of superconductivity above and below *T*_FM_ (Fig. 2 and fig. S5). The weak exchange interaction has previously been suggested by DFT, Mossbauer, and magneto-optic studies in related materials (*23*, *41*–*43*).

An explanation to the field-induced superconductivity mechanism comes by considering both the sizeable dipolar magnetic field exhorted by Eu moments onto the Fe layers and the directional anisotropy of the upper critical field. Using the classical Clausius-Mosotti theory of polarizable media, we determine the dipole field from the stacked infinite planes of fully ordered ferromagnetic Eu moments as B_Eu_ = *m*/3*v* = 0.3T, where *m* = 7 μ_B_ is the magnetization of the Eu moments and *v* = 90 Å^3^ is the volume per moment. This is not an “effective” magnetic field derived from the exchange splitting, but a real field (with respect to the superconducting condensate) that can be screened by Abrikosov vortices (*3*, *4*, *44*, *45*). At 10 K and an applied field of 0.25 T, the XMCD signal saturates at 80% of the 2K XMCD value (fig. S4), suggesting a total dipole field of 0.24 T, in agreement with this estimate. A resistive state is found under zero field, where a net 0.24 T of Eu field is aligned to the *c* axis. Zero resistance is found under an applied field of 0.25 T in plane, which combines with the reoriented Eu moments to give a total 0.49 T of flux in plane. As in other iron-based superconductors (*46*), Eu(Fe_0.88_Co_0.12_)_2_As_2_ has a moderate in- versus out-of-plane *H*_C2_ anisotropy, with γ = *H*_C2,in_/*H*_C2,out_ ≅ 2.1 at *T* = 2 K (fig. S5). As $\gamma >\frac{0.49\;T}{0.25\;T},$and as we expect γ to increase with temperature toward *T*_SC_ (*47*), we can explain the narrow field range of the field-induced superconductivity as due primarily to rotating the Eu moments in plane to take advantage of the higher in-plane critical field. Further, this explains why applied strain does not shift the field range where superconductivity onsets, as strain does not directly tune the Eu magnetic order.

## DISCUSSION

Here, we present field-induced superconductivity between 4 and 10 K, which is enabled with small fields (μ_0_*H* ≤ 0.1 to 0.3 T) and tuned with accessible strain values (|ɛ_xx_| < 0.2%). Our combined XRD, XMCD, and transport measurements show that strain and magnetic field act as independent tuning knobs, with the former affecting the nematic order and Fe antiferromagnetism and the latter affecting the Eu ferromagnetism. These knobs tune the phase diagram analogously to chemical doping, but without introducing additional disorder. The high tunability of this system results from the close competition between the simultaneously coexisting superconducting, nematic, and ferromagnetic phases. In contrast, no field-induced superconductivity has been reported in related Eu-based iron pnictide materials such as EuRbFe_4_As_4_ (*48*) or optimal Ir-doped EuFe_2_As_2_ (*19*), likely due to stronger superconducting order. We anticipate that even higher field-induced superconducting temperatures could be obtained in materials engineered with a perfect balance between higher temperature superconductivity and ferromagnetism.

We further show how the external field-tunable Eu dipole field has a dominant effect on superconductivity when the Eu-Fe exchange splitting is sufficiently weak. This creates a novel mechanism for field-induced superconductivity distinct from the Jaccarino-Peter effect and spin-triplet U-based compounds. This mechanism could likely be present in other systems that exhibit (i) large magnetic moments that are easily field tunable (e.g., *L* = 0 rare earth elements) and (ii) a superconducting order that is dimensionally highly anisotropic [e.g., a van der Waals (vdW) material (*49*–*51*) or at the interface between different materials (*52*)]. This mechanism could arise quite naturally from a vdW heterostructure, with one superconducting layer and one ferromagnetic layer. We note that the apparent first report of field-reentrant superconductivity in a vdW system occurs with stacked thin flakes of antiferromagnetic CrCl_3_ and superconducting NbSe_2_ (*53*), which demonstrates the potential for our proposed mechanism to likewise underlie field-induced superconductivity in 2D materials. Finally, the interaction of ferromagnetism and superconductivity in the presence of very weak exchange splitting has not been widely investigated, yet it may help inform the microscopic understanding of other phenomena. For instance, a recent investigation of EuRbFe_4_As_4_ encountered a spatially modulated superconducting gap, which is presumably triggered by the Eu ferromagnetism (*54*). The proposed explanation of a Fulde, Ferrel, Larkin, and Ovchinnikov (FFLO) phase appears to have some foundational theoretical challenges, which may be resolved by considering the effects of variable and net-cancelling exchange biases, as well as the role the dipole field may play in modulating the superconducting gap.

**Fig. 6.**
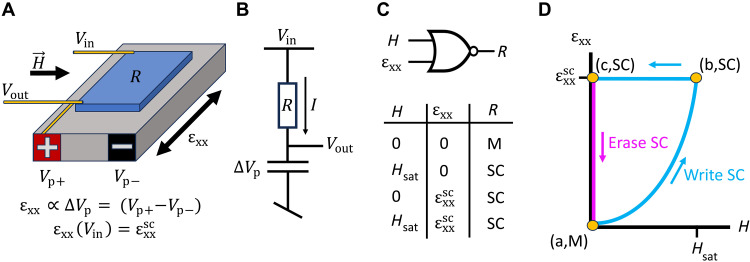
Conceptual approach for a toggle switch using a strain-switchable superconducting spin valve. (**A**) A field-switchable current divider is created by mechanically and electrically connecting a strain-tunable superconducting spin valve (SSV, blue) to a piezo actuator (gray). (**B**) A current I passes through the SSV from the input (*V*_in_) to output (*V*_out_) voltage leads, with *V*_out_ = *V*_in_ − *IR*. The voltage across the piezo is Δ*V*_p_ = *V*_out_. (**C**) Device parameters are chosen so that an applied strain ɛ_xx_(*V*_in_) = $\varepsilon_{\mathrm{xx}}^{\mathrm{SC}}$ and/or an applied field *H* = *H*_sat_ switches the SSV from a metallic state (M, *R* > 0) to a superconducting state (SC, *R* = 0). (**D**) The circuit initializes at point (a) with the SSV in the M state. Write SC (cyan): A writing magnetic field is applied to switch the SSV from the M state to the SC state. As *H* increases to *H*_sat_, *R* reduces to zero, which increases the piezo voltage to Δ*V*_p_ = *V*_in_ and increases the strain to $\varepsilon_{\mathrm{xx}}^{\mathrm{SC}}$ [point (b)]. The additional applied tension maintains the SC state after the writing field is removed [point (c)]. Thus, the device displays memory. Erase SC (magenta): The SSV can be returned to the M state (the written SC state can be erased) by directly discharging the piezo, i.e., by setting *V*_in_ = 0.

Using strain as an additional control switch offers new opportunities to combine superconducting spin valves with the emerging field of “straintronics” (*55*). In Fig. 6, we consider a simple conceptual design for a device that pairs a superconducting spin valve with a piezoelectric substrate. Here, a writing applied magnetic field switches the system into a superconducting state, triggering an increase in tension that maintains the superconductivity after the writing field is removed. Such a device acts as a toggle switch and could be used as a relay, tunable sensor, or memory device. Superconducting spin valves are generally not in situ tunable in their temperature or critical fields; instead, ex situ tuning of the material composition/doping, layer thickness, etc., is required to customize their properties. Thus, the exploration of new routes for in situ strain tuning may lead to new conceptual approaches to superconducting information storage and other technologies.

Another direction for future work is to assess this material’s potential for superconducting spintronics applications by studying the degree of spin polarization and spin-triplet pairing of the supercurrent as it passes through the field-tunable magnetic layers (*5*–*7*). The orientation of the Eu moments can be tuned to an arbitrary direction, allowing control of the spin polarization axis of the emitted spin triplet Cooper pairs, while strain can switch the supercurrent on and off. Finally, the small resistivity just above *T*_0_ has previously been associated with mobile flux vortices making up a spontaneous vortex liquid phase, with zero resistivity indicating the freezing of these vortices (*3*, *4*). An intriguing possibility to explain the enhanced strain tunability of *T*_0_ below *T*_FM_ is that vortices become pinned at nematic domain boundaries (*56*), which can be tuned in number and size with strain. Strain could then be used to switch between a vortex liquid and vortex ice phase at fixed temperature and in the absence (or presence) of field.

## MATERIALS AND METHODS

### Sample preparation

Single-crystal samples of Eu(Fe_0.88_Co_0.12_)_2_As_2_ were grown from a tin flux as described elsewhere (*27*). We used a nonstoichiometric mix ratio of Eu:(Fe_0.85_Co_0.15_):As:Sn of 1:8.5:2:19. This ratio resulted in samples with higher zero-resistance temperatures (*T*_0_) compared to the stoichiometric 1:2:2:20 ratio (fig. S1) (*27*). However, there was substantial sample to sample variability in *T*_0_, which may result from doping inhomogeneity. Samples from a given batch could be found that did not achieve zero resistance at any temperature, while samples 1 and 2 were among the samples with the highest value of *T*_0_ in their batches. The composition of sample 1 was measured by energy-dispersive x-ray spectroscopy (EDX) to be 12% Co-doping, despite a nominal doping of 15%, with a nearly homogeneous value across the sample surface (fig. S1B). The samples were cleaved from large as-grown single-crystal plate and cut along the tetragonal [1 0 0] direction into bars with dimensions ~2 × 0.60 × 0.06 mm. Four gold wires were attached with silver epoxy to measure the inline resistivity ρ_xx_ using a standard four-point measurement and an SR830 lock-in amplifier with 1-mA fixed current. Sample 1 was measured in a Quantum Design PPMS. Sample 2 was measured in x-ray–compatible cryostats at Argonne National Laboratory.

A piezo-actuator uniaxial stress device (Razorbill Instruments, CS-100) was used to provide in situ stress. The built-in capacitance strain gauge was used to determine the nominal strain $\varepsilon_{\mathrm{xx}}^{\mathrm{nom}}$ as in (*31*). Sample chamber constraints prevented the measurement of $\varepsilon_{\mathrm{xx}}^{\mathrm{nom}}$ for data presented in Fig. 5 (C to F). Below the nematic transition (*T*_S_ = 68 K; fig. S3), structural twin domains form along the Fe-Fe bonding direction ([1 1 0]_T_, with lattice constants *a*_or_ and *b*_or_), with orthorhombicity $\varepsilon_{S}=\frac{a_{\mathrm{or}}-b_{\mathrm{or}}}{a_{\mathrm{or}}+b_{\mathrm{or}}}$. Here, we apply stress along the Fe-As bonding direction ([1 0 0]_T_, with lattice constant *a_T_*), resulting in an inline strain $\varepsilon_{\mathrm{xx}}=\frac{\text{Δ}a_{T}}{a_{T,0}}$ and an out-of-plane strain $\varepsilon_{\mathrm{zz}}=\frac{\text{Δ}c}{c_{0}}$. The applied stress thus does not detwin the domains, but instead can tune the magnitude of the nematic order parameter through nonlinear couplings between ɛ_xx_, ɛ_zz_, and ɛ_S_ [see (*29*) and figs. S2 and S3].

After mounting sample 1 on the strain device, a field-, strain-, and temperature-dependent background resistivity of order ρ/ρ_0_ ≈ 1% was present, masking the true entrance into the zero-resistance state. We estimate the field range of field-induced superconductivity from the field range where the resistivity dips below this background (see figs. S6 and S7 for analysis), from which we estimate that field-induced superconductivity occurs in the bulk of the sample up to *T* = 11 K under maximum tension.

Sample 2 was measured during two separate sample cooldowns, with the first yielding the data in Fig. 5 (E and F) and the second for Fig. 5 (C and D). In the first cooldown, the sample reached zero resistance under zero field. With the application of field, a small negative magnetoresistance background was present due to a cryostat wiring issue. After rewiring the cryostat, true zero resistance under field was measured in sample 2 at *T* = 10 K, reported in Fig. 5 (C and D). The data presented in Fig. 5E were corrected to remove this background through a process discussed in the Supplementary Materials; this correction has a minimal (<0.5%) effect on the resistivity for fields near 0.25 T. The data in Fig. 5 (C and D) are the as-measured data.

### XMCD and XRD

XRD measurements were performed at the Advanced Photon Source, beamline 6-ID-B, at Argonne National Laboratory. X-rays of energy 7.6 keV illuminated an area 500 × 500 μm, fully encompassing a cross section of the middle of the crystal where strain transmission is highest. The sample and strain device were mounted on a closed cycle cryostat. Gaussian fits to the tetragonal (1 0 7), (0 0 8), and (1 1 8) reflections were used to determine the lattice constants (*a_T_*), (*c*), and (*a*_or_ and *b*_or_), corresponding to in plane along the stress axis, out of plane, and in plane at 45° to the stress axis, respectively.

XMCD was measured at the Advanced Photon Source beamline 4-ID-D at Argonne National Laboratory. We probed the Eu L_3_ edge using x-rays of 6.97 keV, which measured the spin polarization of the Eu 5d band due primarily to the magnetic moment of the 4f orbital. A superconducting split coil magnet with a large bore was used to apply magnetic field. The sample temperature was controlled using liquid He. XMCD was collected in fluorescence geometry by monitoring the Eu L_α_ line using a four-element Vortex detector integrated with the Xspress module to enable a larger dynamical range. Circularly polarized x-rays were generated using a 180-μm-thick diamond (111) phase plate. Data were corrected for self-absorption. The XMCD spot size illuminates the whole sample width across the *y* direction and is roughly 100 μm wide along the *x* direction (between the transport wires) and probes a depth of about 5 μm. The beam is centered on the middle of the crystal where strain is most transmitted and homogeneous. The incident beam was aligned with the applied magnetic field at an angle of ~10° above parallel to the sample surface (grazing incidence) due to sample chamber constraints. All XMCD data are normalized to the zero-strain, μ_0_*H* > 0.3 T saturated value at *T* = 2 K (fig. S4).

### DFT calculations

The full-potential linearized augmented plane-wave Wien2K package (*39*) has been used for the DFT calculations. We use the Perdew, Burke, and Ernzerhof (*40*) version of the generalized gradient approximation (GGA) to the exchange-correlation functional within DFT. The sphere radii for Eu, Fe, and As are taken as 2.50, 2.29, and 2.18 bohr, respectively. The basis set cutoff parameter RmtKmax = 8.0 was used. The number of *k* points was set to 4500. The crystal structure and magnetic moments on Eu and Fe are illustrated in fig. S8. We set *U* = 9 eV on the Eu atom and did collinear spin-polarized self-consistent calculations in the primitive (not conventional) cell. WIEN2k has a parameter (κ) that tweaks the strength of Hund’s rule coupling. The Hund’s rule coupling is set to normal full strength when κ = 1 and completely switches off when κ = 0. We used this parameter to delineate the two effects mentioned above: The Schrieffer-Wolfe interaction does not depend on the Hund’s rule coupling strength, while the Eu(f)–Eu(d) interaction can be switched off using κ. In fig. S9, we show the band structure for these two values of κ around the Fermi level. The largest splitting near the Fermi level when κ = 1 is about 25 meV along ΓZ-.

## Supplementary Materials

### This PDF file includes:

Supplementary Text Figs. S1 to S14 References
