# Supplementary material for: Strain-switchable field-induced superconductivity
Source: Sci Adv. 2023 Nov 24;9(47):eadj5200. doi: 10.1126/sciadv.adj5200 (PMC10672156; doi:10.1126/sciadv.adj5200)
Supplement: Supplementary file 1 — Supplementary Text Figs. S1 to S14 References [file sciadv.adj5200_sm.pdf]

Science Advances
AAAS

Supplementary Materials for

Strain-switchable field-induced superconductivity

Joshua J. Sanchez *et al.*

Corresponding author: Joshua J. Sanchez, sanchezx@mit.edu; Jiun-Haw Chu, jhchu@uw.edu;
Philip J. Ryan, pryan@anl.gov

Sci. Adv. **9**, eadj5200 (2023)
DOI: 10.1126/sciadv.adj5200

This PDF file includes:

Supplementary Text
Figs. S1 to S14
References

Supplementary Text

I. Crystal Growth

Single crystal samples of $\mathrm{Eu(Fe_{0.85}Co_{0.15})_2As_2}$ were grown from a tin flux as described elsewhere (*27*). We used a nonstoichiometric mix ratio of $\mathrm{Eu:(Fe_{0.85}Co_{0.15}):As:Sn}$ of 1:8.5:2:19. This ratio resulted in samples with higher zero-resistance temperatures ($\mathrm{T_0}$) compared to the stoichiometric 1:2:2:20 ratio (Fig.S1a). However, energy dispersive x-ray spectroscopy (EDX) measurements suggest the Co-doping to be 12% instead of the nominal 15% (Fig.S1b). The superconducting properties of doped $EuFe_2As_2$ depend sensitively on growth methods. For instance, no superconductivity is found for Co-doped samples grown by FeAs flux (*57*). Further investigation of the non-stoichiometric growth conditions is warranted.

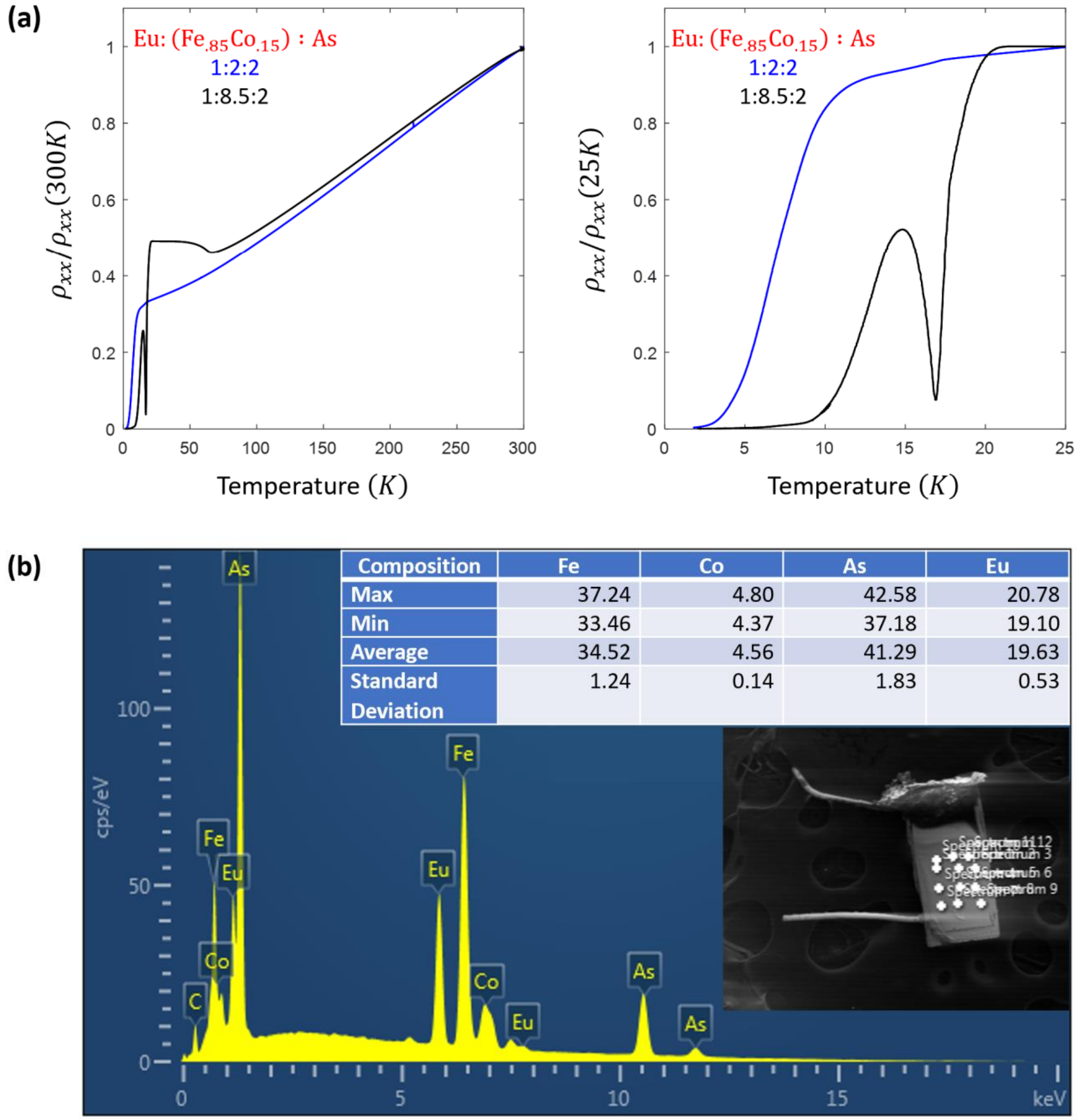

Composition	Fe	Co	As	Eu
Max	37.24	4.80	42.58	20.78
Min	33.46	4.37	37.18	19.10
Average	34.52	4.56	41.29	19.63
Standard Deviation	1.24	0.14	1.83	0.53

Figure S1. Transport and Composition Characterization.

(a) Comparison of resistivity vs temperature for samples grown with an elemental composition that was stoichiometric (blue) and nonstoichiometric (black; sample 2 from main text). **(b)** Sample 1. EDX spectra at 12 points on surface. EDX was performed after removing sample 1 from the strain device after all transport measurements were completed (note the still-attached gold wire). No trace of the Sn flux used in the crystal growth is evident. The nominal composition was Fe=0.85, Co=0.15, while the EDX measured composition was Fe=0.88, Co=0.12.

II. XRD under strain

XRD measurements were performed on sample 2 at the Advanced Photon Source, beamline 6-ID-B, at Argonne National Laboratory. X-rays of energy 7.6 keV illuminated an area 500x500 um, fully encompassing a cross section of the middle of the crystal where strain transmission is highest. The sample and strain device were mounted on a closed cycle cryostat. Gaussian fits to the tetragonal $(1\ 0\ 7)$, $(0\ 0\ 8)$ and $(1\ 1\ 8)$ reflections were used to determine the lattice constants (a_T), (c), and (a_{Or} & b_{Or}, due to the split peak in the twinned state), corresponding to in-plane along the stress axis, out of plane, and in-plane at 45 degrees to the stress axis, respectively.

Figure S2 shows the uniaxial strains $\frac{\Delta a_T}{a_T}$ and $\frac{\Delta c_T}{c_T}$ and the nematic-driven spontaneous orthorhombicity $\varepsilon_{\mathrm{S}} = \frac{a_{Or}-b_{Or}}{a_{Or}+b_{Or}}$ as a function of strain at T=13.5 K. We observe that $\varepsilon_{\mathrm{xx}} = \frac{\Delta a_T}{a_{T,0}}$ is roughly linear to $\varepsilon_{\mathrm{xx}}^{\mathrm{nom}}$ determined from the capacitive strain gauge of the stress device. We find a nearly constant strain transmission of $\frac{\mathrm{d}\varepsilon_{\mathrm{xx}}}{\mathrm{d}\varepsilon_{\mathrm{xx}}^{\mathrm{nom}}}$ =34% across the range of the strains applied. The out of plane uniaxial strain $\varepsilon_{\mathrm{zz}} = \frac{\Delta c}{c_0}$ is surprisingly large compared to ε_{xx} with the Poisson ratio $\nu_{xz} = -\frac{\mathrm{d}\varepsilon_{\mathrm{zz}}}{\mathrm{d}\varepsilon_{\mathrm{xx}}}|_{\varepsilon_{\mathrm{xx}}=0}$ approaching 1.3. A Poisson's ratio greater than unity is unexpected in an isotropic, linear elastic material, and so this large change in planar spacing may result from a substantial magnetostructural response due to the ferromagnetic Eu layers, as well as tuning of the nematic order.

Most importantly, ε_{S} is found to be suppressed by roughly 30% at maximum tension, while being relatively unaffected (or even slightly enhanced) by compression. Given the competition between nematic order and superconductivity, it is clear that the sharp reduction in the resistivity with tension can be attributed (at least in part) by a strain-suppression of the nematic order (see also Fig.S6a). This result is fully in agreement with previous work in Co-doped $BaFe_2As_2$, where tension (compression) applied along the tetragonal [1 0 0] direction resulted in a suppression (enhancement) of the nematic transition temperature (*29*). We observe a similar effect via warming the sample through the nematic transition under either tension or compression (Figure S3). Phenomenologically, the tuning of nematicity with stress applied along the tetragonal [1 0 0] direction results both from the introduction of an orthogonal antisymmetric strain $\left(\varepsilon_{\mathrm{B_{1g}}} = \frac{a_T-b_T}{a_T+b_T}\right)$ which acts to suppress nematicity and ε_{S}, and to a surprisingly large sensitivity to $\varepsilon_{\mathrm{zz}}$ which tunes the unit cell volume despite not breaking any symmetries (see ref. (*29*)).

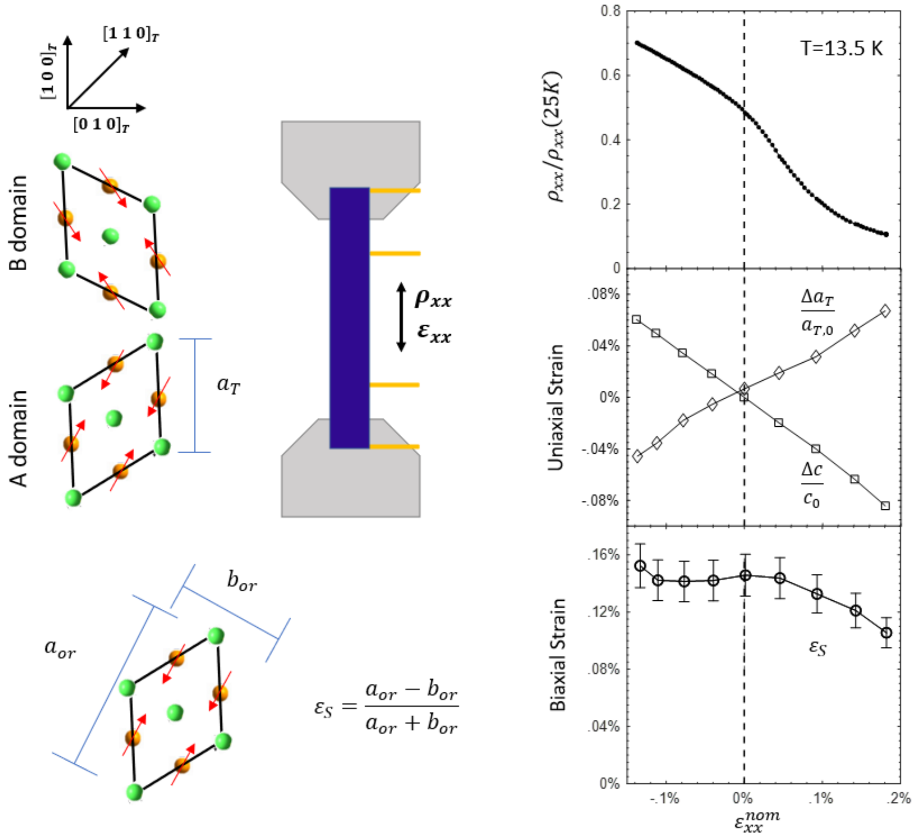

Figure S2. X-ray Diffraction Under Strain.

Sample 2. Fixed temperature (T=13.5K) strain sweep (compressive to tensile) with simultaneous resistivity measurements and XRD measurements of the a_T and c lattice constants (presented normalized by their zero-strain values) and the a_{Or} and b_{Or} lattice constants (presented as the antisymmetric strain ε_S). Error bars on ε_S represent the error propagation of the Gaussian fits to a_{Or} and b_{Or}.

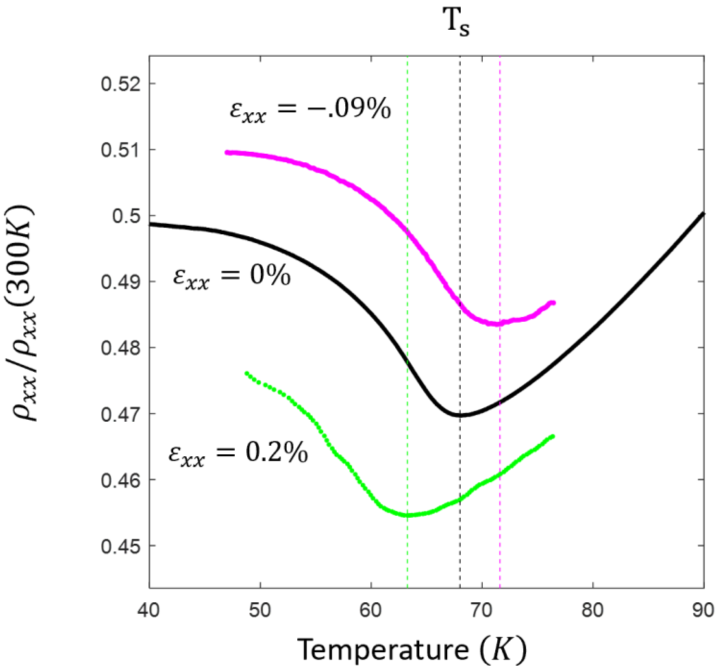

Figure S3. Strain-tuning the nematic transition.

Sample 2. Resistivity vs temperature under zero strain (black, freestanding data from Fig.S1), tension (green) and compression (magenta). Note: the fixed-strain data has been corrected for a ~3 K thermal lag. As such, we do not attempt to make a quantitative assessment of the strain-tuning of the transition temperature, and instead only share this data to show the basic phenomenology of an enhanced (suppressed) nematic transition temperature with compression (tension) in qualitative agreement with past work in Co-doped $BaFe_2As_2$ (*29*).

III. XMCD of 2K result

On sample 2, the first XMCD data were taken after the initial cooldown at zero applied strain at T=2 K and 10 K through a field range of μ_0H=+-1 T. All XMCD data in this work are normalized to this μ_0H>0.3 T, T= 2 K fully saturated XMCD value, which corresponds to the $M \sim 7\ \mu_B$ (μ_0H~0.3 T) fully ordered Eu magnetic moment. The initial XMCD saturation value at T=10K, μ_0H=0.25T is approximately 80% of the 2K saturation value. See Main Text for details of the XMCD measurement.

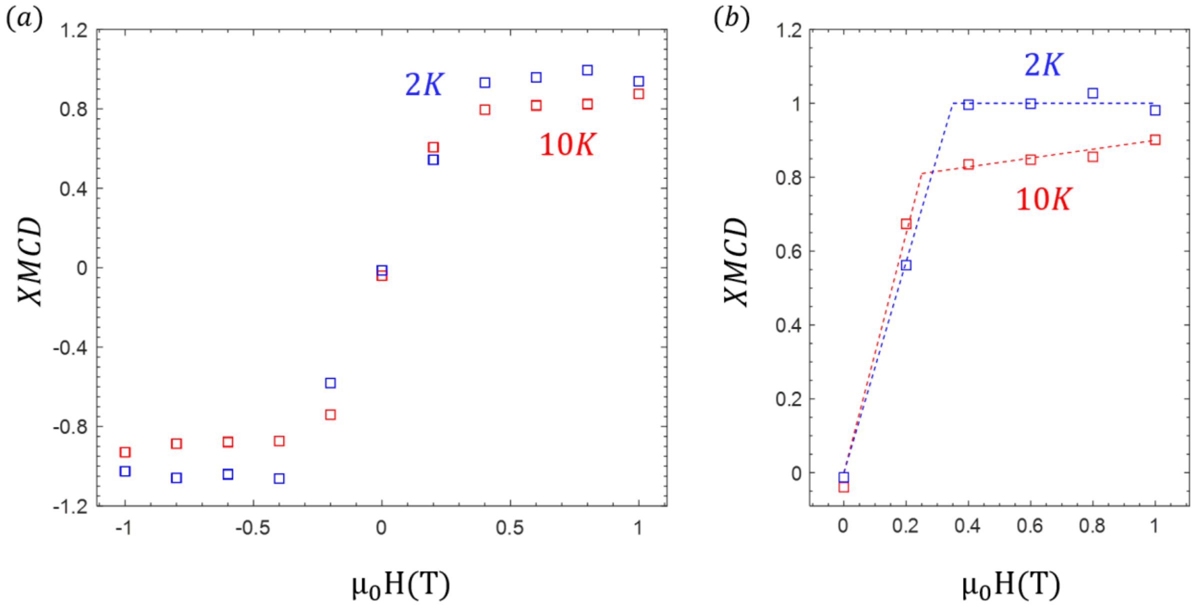

Figure S4. XMCD at 2K and 10K.

Sample 2. XMCD vs field at 2K and 10K. Data in (a) collected from a single field sweep from +1T to -1T. Data in (b) are the normalized difference of the positive and negative field values in (a).

IV. Freestanding magnetoresistance Sample 1

In the freestanding state prior to mounting on the strain cell, sample 1 was cooled through the superconducting and ferromagnetic transitions under zero field (Fig.S5a,b, black), and with an applied field of μ_0H=0.1 T, 0.2 T and 1.0 T either in-plane (Fig.S5a) or out of plane (Fig.S5b). An out of plane field is found to only increase the resistivity, while only lowering the value of T_0. In sharp contrast, an in-plane field is far more detrimental to superconductivity between T_{sc} and T_{FM}, but zero resistance is reached at an enhanced value of T_0=9.0 K for μ_0H=0.2 T, demonstrating field-induced superconductivity.

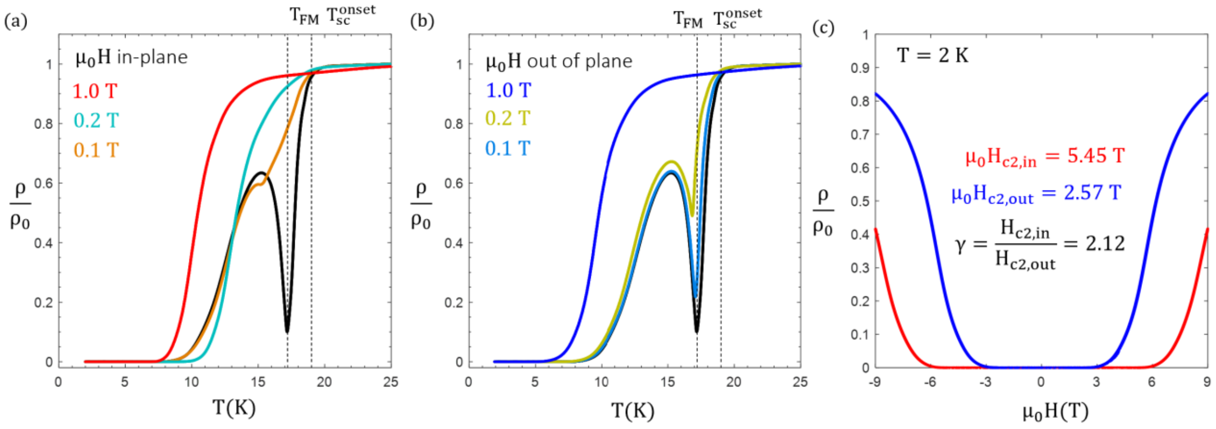

Figure S5. Resistivity for in and out of plane fields.

(a,b) Sample 1. Resistivity vs temperature for zero applied field (black) and μ_0H = 0.1T, 0.2T, and 1T applied in-plane **(a)** and out of plane **(b)**. For μ_0H = 0.2T, applied in-plane, the zero-resistivity temperature rises from T_0 = 7.5K to 9K. **(c)** At T=2 K magnetic field was applied in plane (red) and out of plane (blue) to extract the upper critical fields H_{c2} for each direction, yielding an anisotropy term $\gamma \cong 2.1$.

V. Assessing the nonzero background resistance of Sample 1

Extensive resistivity measurements of sample 1 were made under different temperature, field and strain states. After mounting sample 1 on the strain device, a field, strain and temperature dependent background resistivity of order $\rho \approx 0.01\%$ was present, masking the true entrance into the zero-resistance state (Fig.S6). This may be due to a small volume of the sample which buckled under strain and thus behaves as if heavily-compressed, effectively raising its respective value of T_0 while still being highly strain, field and temperature dependent.

Here we describe the workaround to this issue. Prior to mounting on the strain device, the sample reached zero resistance at $T_0 = 7.5$ K. In Figure S6, the orange trace is under small tension ($\varepsilon_{xx} = 0.04\%$) and has a lower resistivity than the freestanding trace at all temperatures above T=9K, indicating an enhancement to the superconductivity. Below T=9 K, the orange trace has a higher resistivity and never reaches zero. The sample should be expected to reach zero resistance at higher temperature under tension, as is observed in sample 2. At T=7.5K, the orange trace should already be in a zero-resistance state, but instead has a value of approximately $\rho/\rho_0 = 1\%$. We thus use this value as a conservative estimate for the temperature and field entrance into the true zero resistance state. We determine the value of T_0 under fixed strain as $T_0(\varepsilon_{xx} = -.19\%) = 4K$, $T_0(\varepsilon_{xx} = .04\%) = 7.5K$, and $T_0(\varepsilon_{xx} = .20\%) = 10.3K$. We note that the data presented in Fig.S6 covers the full range of tensile and compressive strains we could apply with our device.

In Figure S7 we show the resistivity vs applied in-plane magnetic field data at several temperatures under $\varepsilon_{xx} = -.19\%$ (a) and $\varepsilon_{xx} = .20\%$ (b). The field values where the resistivity crosses $\rho/\rho_0 = 0.01\%$ are indicated by square markers, and these values are used to define the field-induced superconductivity phase space in the Main Text Figure 4.

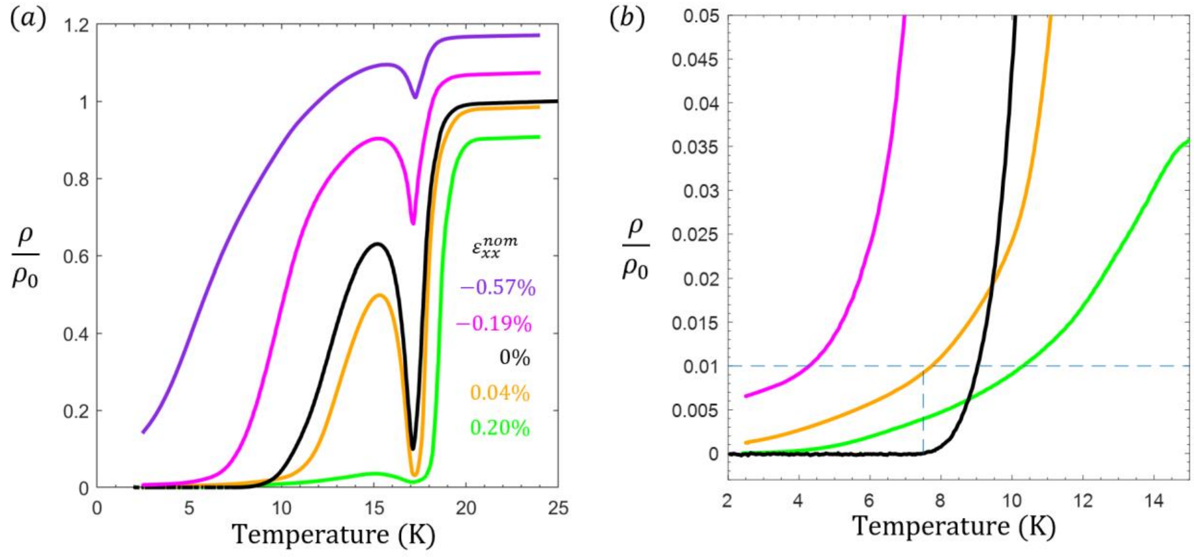

Figure S6. Strain-tuned superconducting transition.

(a) Sample 1. Resistivity vs temperature at two compressive and two tensile strain values, compared to freestanding value (black). **(b)** The same data zoomed to observe low resistivity values.

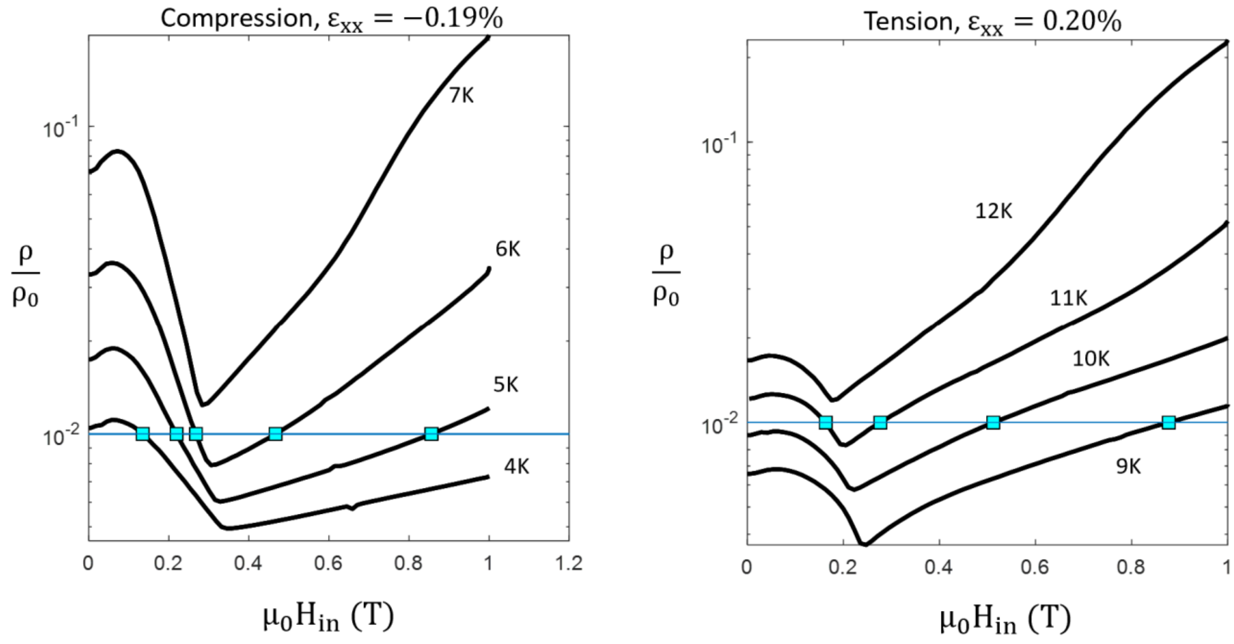

Figure S7. Small nonzero background resistivity.

Sample 1. Resistivity vs applied in-plane magnetic field under compression (left) and tension (right). The ρ/ρ_0>0.01 line marks the cutoff for estimated entrance into bulk zero-resistance state.

VI. Density Functional Theory Analysis of Eu-Fe exchange interactions

A puzzle is presented by the finding that an in-plane field is far more detrimental to superconductivity than an out of plane field above the Eu ferromagnetic ordering ($T_{SC} > T > T_{FM}$), while it promotes superconductivity below the maximum of the reentrant resistivity (Fig.S5). A plausible explanation can be given by considering the two competing effects of Eu moments on the Fe spin polarization. First, when Eu moments are aligned in-plane, the direct hybridization between Fe 3d states with Eu 4f states creates a spin-polarizing exchange bias, which pushes Eu-aligned Fe spin states at the Fermi surface up in energy and anti-aligned states down (Schrieffer-Wolfe interaction). This induces a direct exchange splitting $\Delta_d \sim t_{Fe(d)\text{-}Eu(f)}^2/(E_F\text{-}E_{Eu(f)})$, where the induced Fe polarization is *antiparallel* to that of the Eu ions. Second, there is a competing interaction due to hybridization between the Fe bands and the empty Eu d-bands. Since the latter are coupled to Eu 4f states though Hund rule's coupling, this effect leads to a net polarization of Fe that is *parallel* to Eu moments. A net polarization of Fe moments in *either* direction suppresses Cooper pairing.

In order to estimate these effects quantitatively, we have performed DFT calculations using the Wien2K package (*39*, *40*) to characterize these two effects. We find that both show high sensitivity to the Hubbard U on Eu sites, and as both are small and opposite in sign, their sum can be of either sign, or even of different signs in different bands. Below the Eu ferromagnetic transition, these two effects seem to largely cancel, and the dipole-field mechanism is dominant. Above the transition, the Eu moments do not fully saturate at small applied field, and the (accidental) cancelation between the two electronic effects will be lifted. This causes the itinerant Fe moments to gain a net magnetization for Eu moments aligned in-plane, which yields the stronger suppression of superconductivity with in-plane field. While we cannot calculate this exchange field with the required accuracy, given the strong dependence on the applied Hubbard correction, we can estimate the order of magnitude of the effect. We find that for fully in-plane polarized Eu moments (with $m = 7\ u_B$) and a variable U, the exchange interaction is within ±10 meV. This is far larger than the anticipated superconducting gap of the order of 2 meV (given T_{SC} = 19K), and so the scale of the effect – barring the above-discussed accidental cancellation – is on the right order to suppress superconductivity for the in-plane field. Meanwhile, for an out-of-plane applied field, Eu and Fe moments are perpendicular and the exchange splitting is only second order in the exchange field, resulting in a much weaker Eu-driven polarization of Fe spins and a far milder suppression of superconductivity. This explains why the effect of magnetic field shows the opposite anisotropy above and below the ferromagnetic ordering temperature of Eu.

Here we discuss the details of our calculations. We have used density functional theory (DFT) to characterize the effects of the Eu moments on the antiferromagnetically-ordered Fe moments via different exchange interactions, in order to estimate the band effects of Eu magnetism on Fe-origin superconductivity. We directly observe that both parallel and antiparallel Eu-Fe alignment effects are present, which appear to largely cancel out at low temperature.

We also show projected Fe d, Eu f and Eu d bands for κ=0 in Fig. S10. In the κ=0 case, Fe1 and Fe2 have equivalent small moments that have the same direction as the Eu moment, so we do not differentiate Fe1 and Fe2 in projected band. We can clearly see Fe d spin up bands pushed up above the Eu f bands, and this effect is larger closer to Eu f bands. Another effect is a non-negligible Fe d spin up contribution in the Eu f bands.

Comparing calculations with the full Hund's rule coupling (κ=1), where both Schrieffer-Wolfe Eu(f)-Fe(d) antiferromagnetic coupling and Hund's rule Eu(f)-Eu(d) ferromagnetic coupling is included, and those with κ=0, where only the former is operative, we see that (a) Eu(f)-Fe(d) is indeed antiferromagnetic (the red bands are always below the blue ones), and rather large for some bands at the Fermi level, while the competing Eu(f)-Eu(d) interactions is largely cancelling it for κ=1, and this cancellation is, fortuitously, nearly complete right at the Fermi level (while at ~0.2 eV below or above it becomes large, up to 100 meV). This confirms our conjecture of a fragile character of this cancellation.

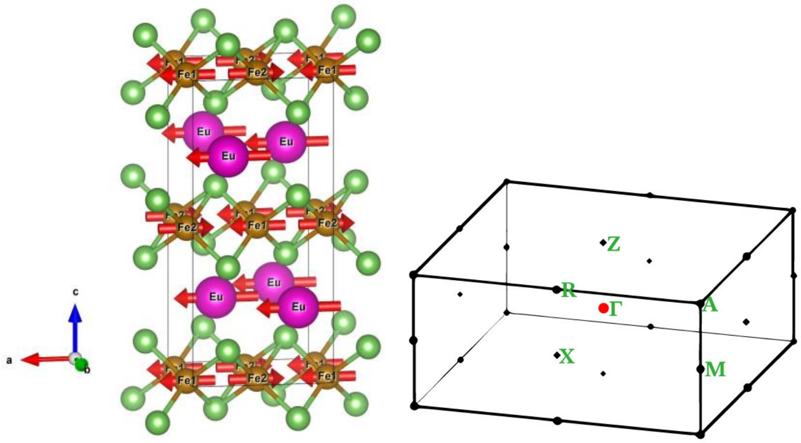

Figure S8. DFT calculations: Brillouin Zone.

Conventional structure, with a = 5.5372Å, b = 5.5052Å, c = 12.0572Å. Eu atoms are ferromagnetically ordered along the easy axis of the Fe antiferromagnetic order. High symmetry points of corresponding BZ are shown on right.

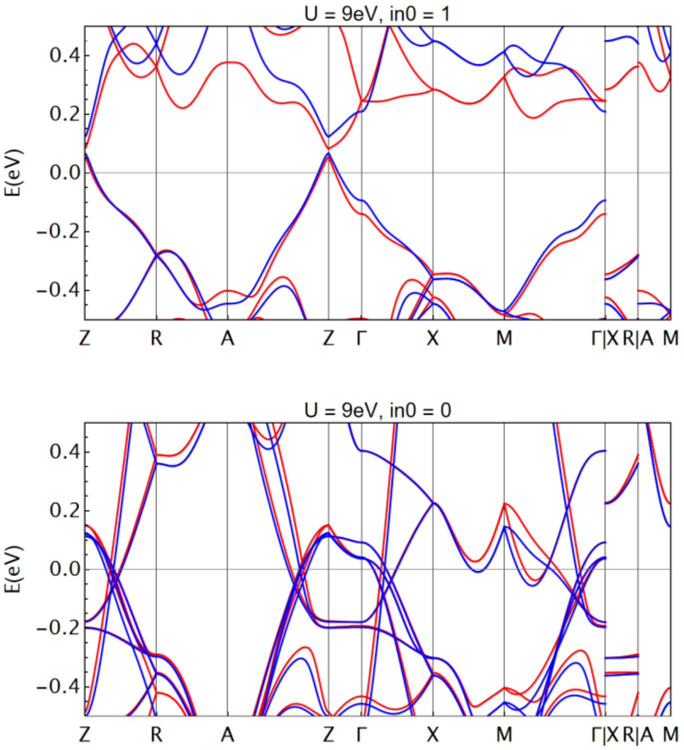

Figure S9. DFT calculations: Band Structure.

Band structure of U=9eV for κ=0 and 1. Red bands for spin up (aligned to Eu moments) and blue bands for spin down (anti-aligned to Eu moments).

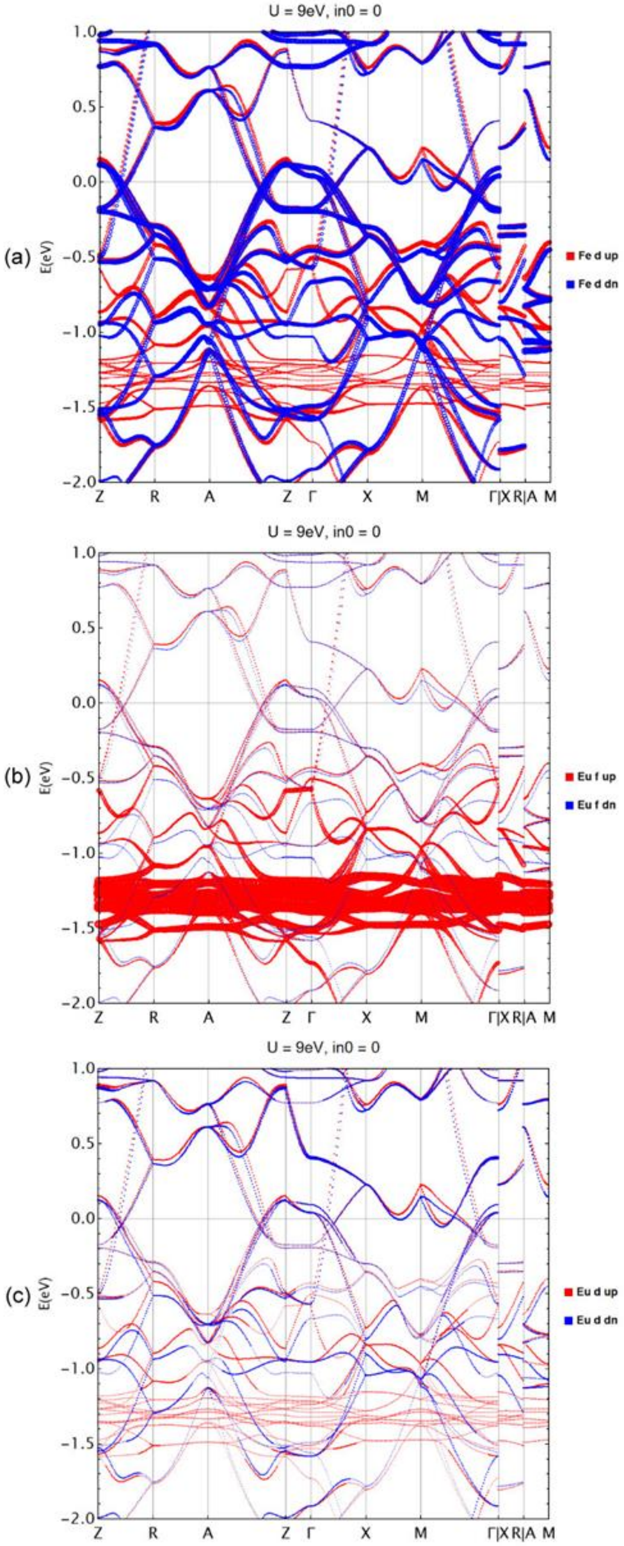

Figure S10. DFT calculations: Element-specific band structure.

Projected bands of (a) Fe d, (b) Eu f and (c) Eu d.

VII. Additional transport and XMCD data and details for Sample 2.

In the initial measurement of sample 2 in the x-ray compatible cryostat, zero resistance was obtained at zero applied magnetic field. During the measurement of the resistivity data in Fig.5E, it was noted that a small negative quadratic magnetoresistance background was present, causing the fully superconducting phase to appear to have negative resistance. We found that this was due to inductive noise from the wiring within the cryostat, which had not been optimized for low resistivity measurements. We fixed this wiring issue and reran the same sample under nearly-identical temperature, field and strain conditions to produce the data in Fig.5C,D, which did not show any negative magnetoresistance and which has not been corrected in any way. The data presented in Fig.5E was corrected to remove this background. To do so, we fit a quadratic function to the negative magnetoresistance of the sample in the highest-tension fully-superconducting state (Fig.S11, purple). This fit had an R^2 value of 0.97. The background had the most noticeable effect (<4%) at high field and only a very small effect (<0.5%) in the vicinity of 0.25T, where the minimum of the real sample magnetoresistance coincides with the full saturation of the Eu moments. Thus, we cannot say with certainty whether the +100V resistivity curve reaches zero resistance or not, while the analogous data in Fig.5C,D unambiguously does.

In Fig.S12, we present the complete set of (self-absorption corrected) x-ray absorption near-edge structure (XANES) and XMCD measurements performed in sample 2 used to produce Fig.5F. It is seen that strain does not visibly affect either quantity, confirming that strain has a minimal effect on the Eu magnetic order.

In Fig.S13, we present XMCD data at T=15K under large compression, and compare it to T=10K data at equivalent compression. Note that the XMCD self-absorption correction was not performed for the 15K data, as a necessary wide energy scan measurement was not performed at this temperature. To better compare it with the 10K data, we show here the 10K XMCD without the self-absorption correction, which is only subtly different from the version presented in Fig.S12.

In Fig.S14, we show resistivity vs device voltage at T=7K, below the zero-strain superconducting zero-resistance temperature, at both zero field and at the optimal in-plane field of 0.26T. We compare this with the main text figure 5C, which is an equivalent data set at T=10K. At T=7K, zero resistance is found over a wide strain range.

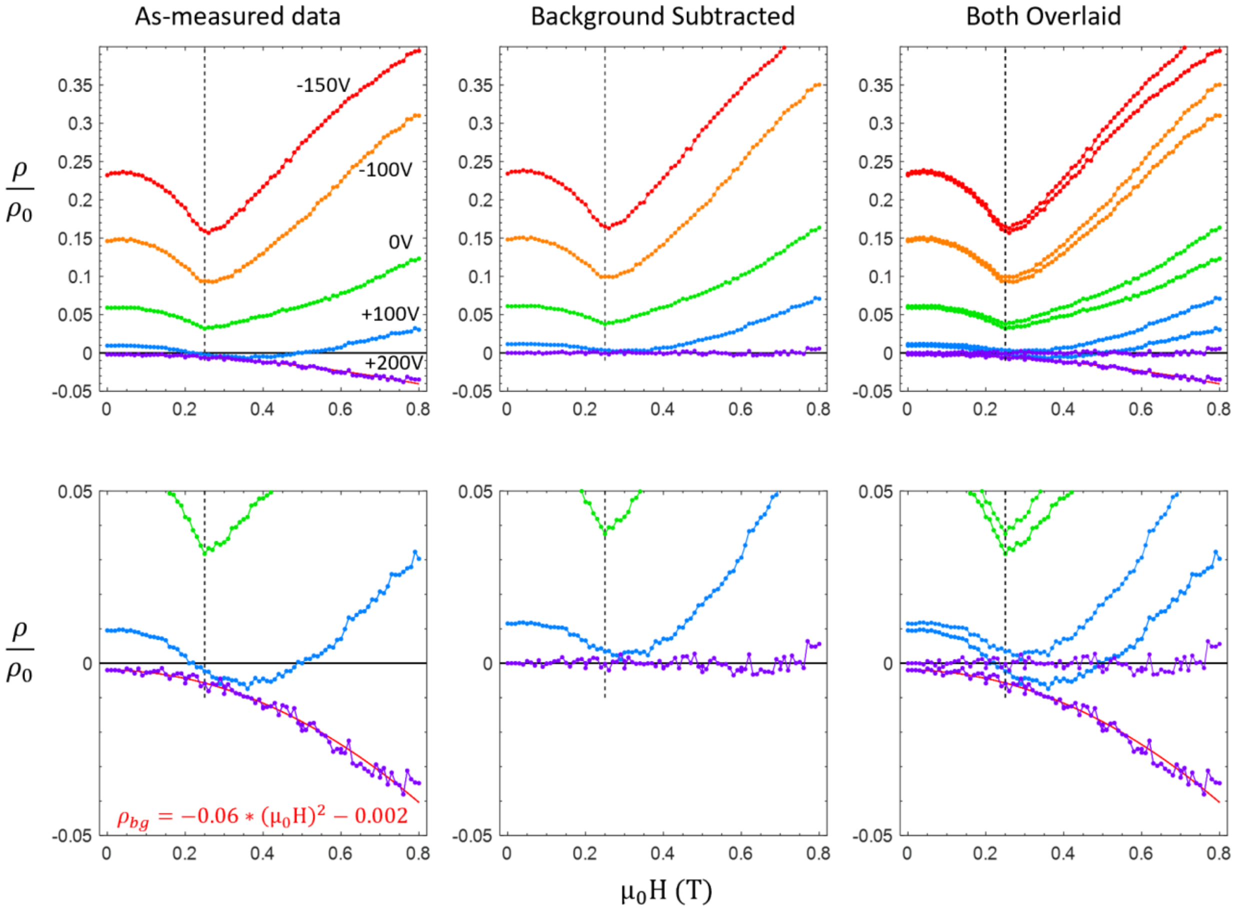

Figure S11. Field-dependent magnetoresistance background subtraction.

Resistivity vs field for data in main text Fig.5E. Bottom plots are zoomed in to small resistance range. Negative quadratic magnetoresistance (red fit line) present as a background signal during the data collection. This background was subtracted from all 5 resistivity traces. The overlay of pre- and post-background subtracted data shows that this background has a minimal effect on the resistivity near 0.25T. Thus, it is clear that the minimum of the magnetoresistance corresponds to the full in-plane polarization of Eu moments.

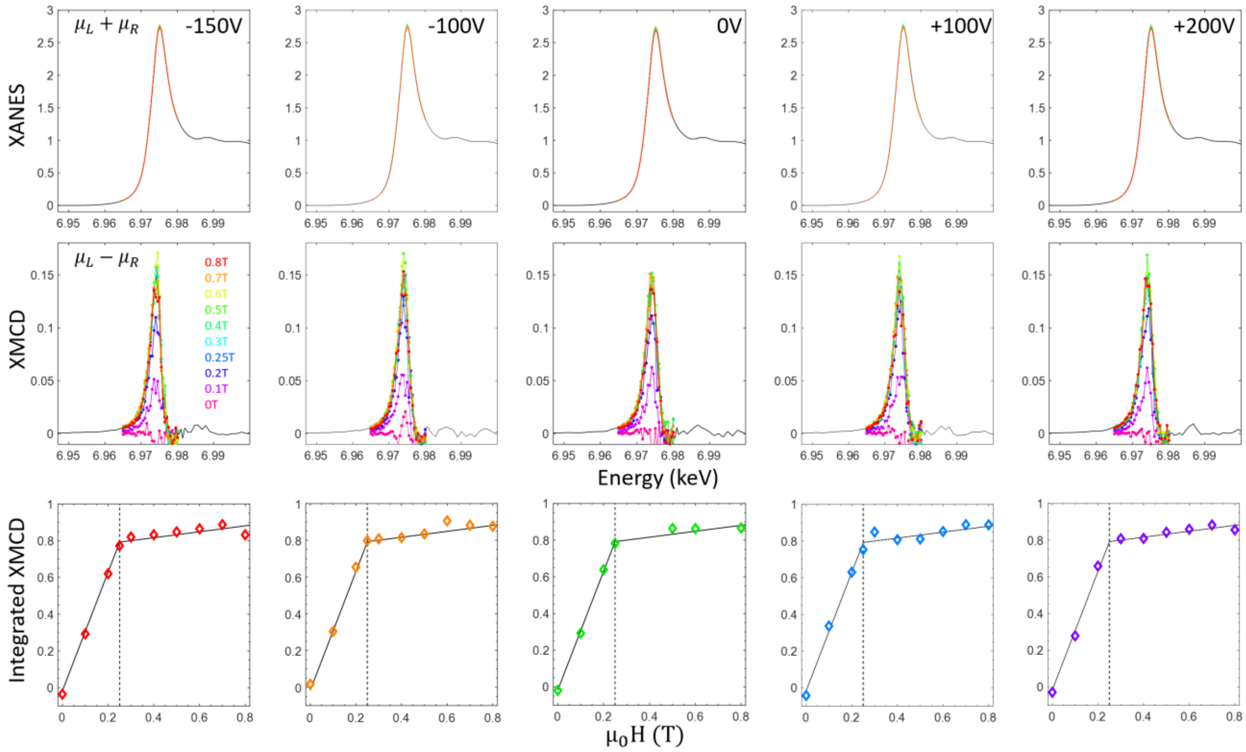

Figure S12. XMCD full spectra.

Sample 2. X-ray spectroscopy under strain and field at T=10K from main text Fig.5F. μ_L and μ_R refer to left and right circular polarized light. Top: X-ray absorption near edge spectrum (XANES). Middle: X-ray magnetic circular dichroism (XMCD). Bottom: sum of the XMCD data, normalized to the T=2K XMCD signal (see Fig.S4). Lines are a guide to the eye and are the same in all figures. Black traces in top/middle are wider energy scans at 0.8T applied field. Colored scans are narrow energy scans at field values 0-0.8T. Bottom plots are color coded as in Fig.5F. Left to right is strain from compression to tension. The field-tuned XANES are superimposed in top figures and are nearly indistinguishable. Further, strain tuning has virtually no effect on the XANES. The XMCD figures change only subtly with strain at same equal field values.

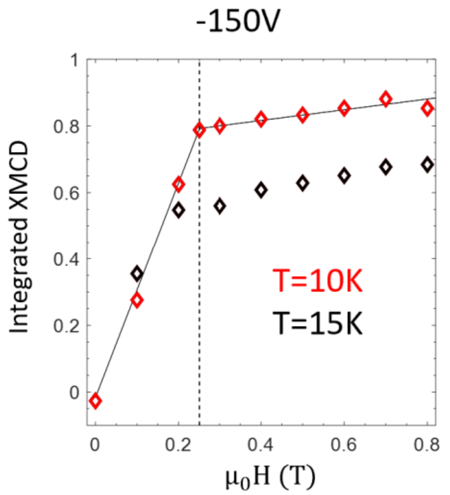

Figure S13. XMCD at 10K and 15K.

Sample 2. XMCD vs field at 15K and 10K under large compression (-150V). The Eu magnetic moment at 15K is clearly reduced relative to 10K, and correspondingly reaches saturation at a lower field.

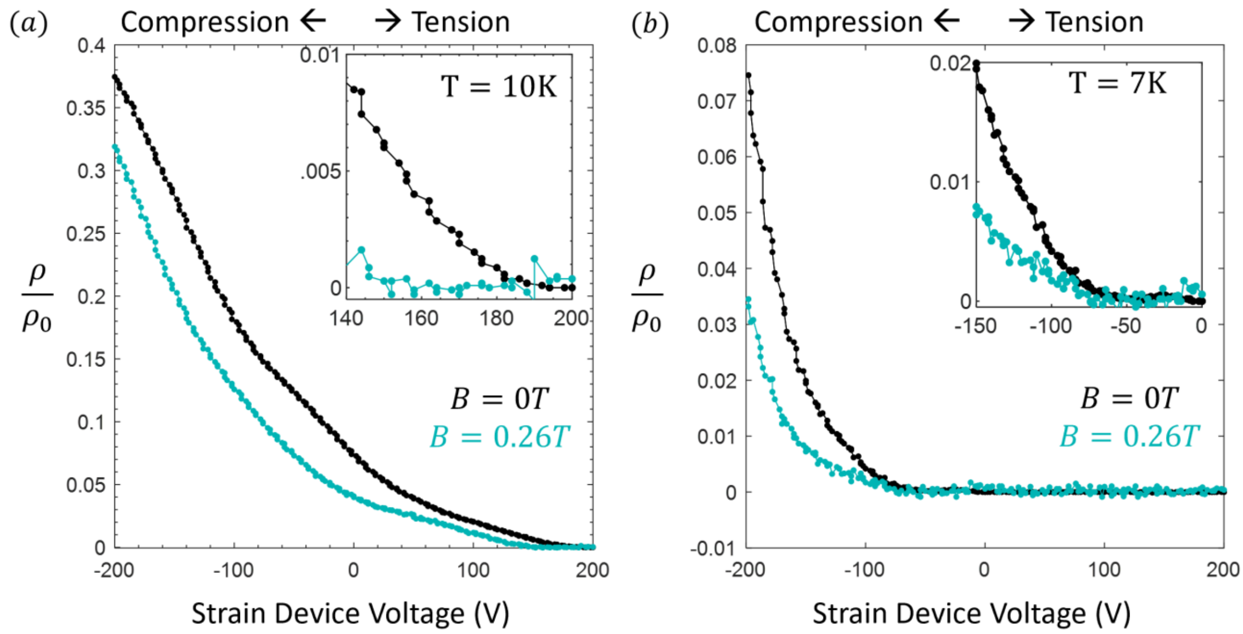

Figure S14. Strain and field tunable resistivity above and below the zero-resistance temperature.

Sample 2. Resistivity vs strain device voltage at (a) T=10K and (b) T=7K. Note that at T=10K, tension must be applied to induce zero resistance, while at T=7K, compression must be applied to return to a nonzero resistance state. In both cases, a near-optimal applied magnetic field reduces the resistivity at all voltages.

REFERENCES AND NOTES

1. D. N. Basov, R. D. Averitt, D. Hsieh, Towards properties on demand in quantum materials. *Nat. Mater.* **16**, 1077–1088 (2017).

2. S. Nandi, W. T. Jin, Y. Xiao, Y. Su, S. Price, D. K. Shukla, J. Strempfer, H. S. Jeevan, P. Gegenwart, Th. Brückel, Coexistence of superconductivity and ferromagnetism in P-doped $EuFe_2As_2$. *Phys. Rev. B* **89**, 014512 (2014).

3. W.-H. Jiao, Q. Tao, Z. Ren, Y. Liu, G.-H. Cao, Evidence of spontaneous vortex ground state in an iron-based ferromagnetic superconductor. *npj Quantum Mater.* **2**, 50 (2017).

4. V. S. Stolyarov, I. S. Veshchunov, S. Yu. Grebenchuk, D. S. Baranov, I. A. Golovchanskiy, A. G. Shishkin, N. Zhou, Z. Shi, X. Xu, S. Pyon, Y. Sun, W. Jiao, G.-H. Cao, L. Ya. Vinnikov, A. A. Golubov, T. Tamegai, A. I. Buzdin, D. Roditchev, Domain Meissner state and spontaneous vortex-antivortex generation in the ferromagnetic superconductor $EuFe_2(As_{0.79}P_{0.21})_2$. *Sci. Adv.* **4**, eaat1061 (2018).

5. J. Linder, J. W. A. Robinson, Superconducting spintronics. *Nat. Phys.* **11**, 307–315 (2015).

6. G. D. Simoni, E. Strambini, J. S. Moodera, F. S. Bergeret, F. Giazotto, Toward the absolute spin-valve effect in superconducting tunnel junctions. *Nano Lett.* **18**, 6369–6374 (2018).

7. M. G. Flokstra, N. Satchell, J. Kim, G. Burnell, P. J. Curran, S. J. Bending, J. F. K. Cooper, C. J. Kinane, S. Langridge, A. Isidori, N. Pugach, M. Eschrig, H. Luetkens, A. Suter, T. Prokscha, S. L. Lee, Remotely induced magnetism in a normal metal using a superconducting spin-valve. *Nat. Phys.* **12**, 57–61 (2016).

8. B. Li, N. Roschewsky, B. A. Assaf, M. Eich, M. Epstein-Martin, D. Heiman, M. Münzenberg, J. S. Moodera, Superconducting spin switch with infinite magnetoresistance induced by an internal exchange field. *Phys. Rev. Lett.* **110**, 097001 (2013).

9. H. W. Meul, C. Rossel, M. Decroux, Ø. Fischer, G. Remenyi, A. Briggs, Observation of magnetic-field-induced superconductivity. *Phys. Rev. Lett.* **53**, 497–500 (1984).

10. J. Cors, R. Baíllif, M. G. Karkut, M. Decroux, Ø. Fischer, U. Welp, G. Bruls, Observation of magnetic-field-induced superconductivity in Se-doped EuMo6S8 under pressure. *Europhys. Lett.* **3**, 635–641 (1987).

11. F. Lévy, I. Sheikin, B. Grenier, A. D. Huxley, Magnetic field-induced superconductivity in the ferromagnet URhGe. *Science* **309**, 1343–1346 (2005).

12. P. T. Yang, Z. Y. Liu, K. Y. Chen, X. L. Liu, X. Zhang, Z. H. Yu, H. Zhang, J. P. Sun, Y. Uwatoko, X. L. Dong, K. Jiang, J. P. Hu, Y. F. Guo, B. S. Wang, J.-G. Cheng, Pressured-induced superconducting phase with large upper critical field and concomitant enhancement of antiferromagnetic transition in $EuTe_2$. *Nat. Commun.* **13**, 2975 (2022).

13. G. Knebel, W. Knafo, A. Pourret, Q. Niu, M. Vališka, D. Braithwaite, G. Lapertot, M. Nardone, A. Zitouni, S. Mishra, I. Sheikin, G. Seyfarth, J.-P. Brison, D. Aoki, J. Flouquet, Field-reentrant superconductivity close to a metamagnetic transition in the heavy-fermion superconductor UTe_2. *J. Physical Soc. Japan* **88**, 063707 (2019).

14. S. Ran, S. R. Saha, I. L. Liu, D. Graf, J. Paglione, N. P. Butch, Expansion of the high field-boosted superconductivity in UTe_2 under pressure. *npj Quantum Mater.* **6**, 75 (2021).

15. T. Konoike, S. Uji, T. Terashima, M. Nishimura, S. Yasuzuka, K. Enomoto, H. Fujiwara, B. Zhang, H. Kobayashi, Magnetic-field-induced superconductivity in the antiferromagnetic organic superconductor κ−$(BETS)_2FeBr_4$. *Phys. Rev. B* **70**, 094514 (2004).

16. K. Hiraki, H. Mayaffre, M. Horvatić, C. Berthier, S. Uji, T. Yamaguchi, H. Tanaka, A. Kobayashi, H. Kobayashi, T. Takahashi, ^{77}Se NMR evidence for the jaccarino–Peter mechanism in the field induced superconductor, λ-$(BETS)_2FeCl_4$. *J. Physical Soc. Japan* **76**, 124708 (2007).

17. S. Jiang, H. Xing, G. Xuan, Z. Ren, C. Wang, Z. Xu, G. Cao, Superconductivity and local-moment magnetism in $Eu(Fe_{0.89}Co_{0.11})_2As_2$. *Phys. Rev. B* **80**, 184514 (2009).

18. U. B. Paramanik, P. L. Paulose, S. Ramakrishnan, A. K. Nigam, C. Geibel, Z. Hossain, Magnetic and superconducting properties of Ir-doped $EuFe_2As_2$. *Supercond. Sci. Technol.* **27**, 075012 (2014).

19. W.-H. Jiao, H.-F. Zhai, J.-K. Bao, Y.-K. Luo, Q. Tao, C.-M. Feng, Z.-A. Xu, G.-H. Cao, Anomalous critical fields and the absence of Meissner state in $Eu(Fe_{0.88}Ir_{0.12})_2As_2$ crystals. *New J. Phys.* **15**, 113002 (2013).

20. H. S. Jeevan, D. Kasinathan, H. Rosner, P. Gegenwart, Interplay of antiferromagnetism, ferromagnetism, and superconductivity in $EuFe_2(As_{1-x}P_x)_2$ single crystals. *Phys. Rev. B.* **83**, 054511 (2011).

21. S. Zapf, M. Dressel, Europium-based iron pnictides: A unique laboratory for magnetism, superconductivity and structural effects. *Rep. Prog. Phys.* **80**, 016501 (2017).

22. A. Baumgartner, D. Neubauer, S. Zapf, A. V. Pronin, W. H. Jiao, G. H. Cao, M. Dressel, Reentrant phases in electron-doped $EuFe_2As_2$: Spin glass and superconductivity. *Phys. Rev. B* **95**, 174522 (2017).

23. I. Nowik, I. Felner, Z. Ren, G. H. Cao, Z. A. Xu, Coexistence of ferromagnetism and superconductivity: Magnetization and Mössbauer studies of $EuFe_2(As_{1-x}P_x)_2$. *J. Phys. Condens. Matter* **23**, 065701 (2011).

24. W.-H. Jiao, J.-K. Bao, Q. Tao, H. Jiang, C.-M. Feng, Z.-A. Xu, G.-H. Cao, Evolution of superconductivity and ferromagnetism in $Eu(Fe_{1-x}Ru_x)_2As_2$. *J. Phys. Conf. Ser.* **400**, 022038 (2012).

25. W. T. Jin, Y. Xiao, Z. Bukowski, Y. Su, S. Nandi, A. P. Sazonov, M. Meven, O. Zaharko, S. Demirdis, K. Nemkovski, K. Schmalzl, L. M. Tran, Z. Guguchia, E. Feng, Z. Fu, Th. Brückel, Phase diagram of Eu magnetic ordering in Sn-flux-grown $Eu(Fe1-xCox)_2As_2$ single crystals. *Phys. Rev. B* **94**, 184513 (2016).

26. W. T. Jin, W. Li, Y. Su, S. Nandi, Y. Xiao, W. H. Jiao, M. Meven, A. P. Sazonov, E. Feng, Y. Chen, C. S. Ting, G. H. Cao, Th. Brückel, Magnetic ground state of superconducting $Eu(Fe_{0.88}Ir_{0.12})_2As_2$: A combined neutron diffraction and first-principles calculation study. *Phys. Rev. B* **91**, 064506 (2015).

27. V. H. Tran, T. A. Zaleski, Z. Bukowski, L. M. Tran, A. J. Zaleski, Tuning superconductivity in $Eu(Fe_{0.81}Co_{0.19})_2As_2$ with magnetic fields. *Phys. Rev. B* **85**, 052502 (2012).

28. A. Löhle, A. Baumgartner, S. Zapf, M. Dressel, W. H. Jiao, G. H. Cao, Effects of pressure and magnetic field on the reentrant superconductor $Eu(Fe_{0.93}Rh_{0.07})_2As_2$. *Phys. Rev. B.* **95**, 195146 (2017).

29. M. S. Ikeda, T. Worasaran, J. C. Palmstrom, J. A. W. Straquadine, P. Walmsley, I. R. Fisher, Symmetric and antisymmetric strain as continuous tuning parameters for electronic nematic order. *Phys. Rev. B* **98**, 245133 (2018).

30. P. Malinowski, Q. Jiang, J. J. Sanchez, J. Mutch, Z. Liu, P. Went, J. Liu, P. J. Ryan, J.-W. Kim, J.-H. Chu, Suppression of superconductivity by anisotropic strain near a nematic quantum critical point. *Nat. Phys.* **16**, 1189–1193 (2020).

31. J. J. Sanchez, P. Malinowski, J. Mutch, J. Liu, J.-W. Kim, P. J. Ryan, J.-H. Chu, The transport–Structural correspondence across the nematic phase transition probed by elasto X-ray diffraction. *Nat. Mater.* **20**, 1519–1524 (2021).

32. X. Chen, S. Maiti, R. M. Fernandes, P. J. Hirschfeld, Nematicity and superconductivity: Competition versus cooperation. *Phys. Rev. B* **102**, 184512 (2020).

33. A. Akbari, P. Thalmeier, I. Eremin, Evolution of the multiband Ruderman–Kittel–Kasuya–Yosida interaction: Application to iron pnictides and chalcogenides. *New J. Phys.* **15**, 033034 (2013).

34. J. J. Sanchez, G. Fabbris, Y. Choi, Y. Shi, P. Malinowski, S. Pandey, J. Liu, I. I. Mazin, J.-W. Kim, P. Ryan, J.-H. Chu, Strongly anisotropic antiferromagnetic coupling in $EuFe_2As_2$ revealed by stress detwinning. *Phys. Rev. B* **104**, 104413 (2021).

35. S. Zapf, C. Stingl, K. W. Post, J. Maiwald, N. Bach, I. Pietsch, D. Neubauer, A. Löhle, C. Clauss, S. Jiang, H. S. Jeevan, D. N. Basov, P. Gegenwart, M. Dressel, Persistent detwinning of iron-pnictide $EuFe_2As_2$ crystals by small external magnetic fields. *Phys. Rev. Lett.* **113**, 227001 (2014).

36. J. Maiwald, I. I. Mazin, P. Gegenwart, Microscopic theory of magnetic detwinning in iron-based superconductors with large-spin rare earths. *Phys. Rev. X* **8**, 011011 (2018).

37. V. Jaccarino, M. Peter, Ultra-high-field superconductivity. *Phys. Rev. Lett.* **9**, 290–292 (1962).

38. L. Balicas, J. S. Brooks, K. Storr, S. Uji, M. Tokumoto, H. Tanaka, H. Kobayashi, A. Kobayashi, V. Barzykin, L. P. Gor'kov, Superconductivity in an organic insulator at very high magnetic fields. *Phys. Rev. Lett.* **87**, 067002 (2001).

39. P. Blaha, K. Schwarz, F. Tran, R. Laskowski, G. K. H. Madsen, L. D. Marks, WIEN2k: An APW+lo program for calculating the properties of solids. *J. Chem. Phys.* **152**, 074101 (2020).

40. J. P. Perdew, K. Burke, M. Ernzerhof, Generalized gradient approximation made simple. *Phys. Rev. Lett.* **77**, 3865–3868 (1996).

41. I. Nowik, I. Felner, Z. Ren, G. H. Cao, Z. A. Xu, ^{57}Fe and ^{151}Eu Mössbauer spectroscopy and magnetization studies of $Eu(Fe_{0.89}Co_{0.11})_2As_2$ and $Eu(Fe_{0.9}Ni_{0.1})_2As_2$. *New J. Phys.* **13**, 023033 (2011).

42. H. S. Jeevan, Z. Hossain, D. Kasinathan, H. Rosner, C. Geibel, P. Gegenwart, Electrical resistivity and specific heat of single-crystalline $EuFe_2As_2$: A magnetic homologue of $SrFe_2As_2$. *Phys. Rev. B* **78**, 052502 (2008).

43. A. Pogrebna, T. Mertelj, N. Vujičić, G. Cao, Z. A. Xu, D. Mihailovic, Coexistence of ferromagnetism and superconductivity in iron based pnictides: A time resolved magnetooptical study. *Sci. Rep.* **5**, 7754 (2015).

44. V. L. Ginzburg, Ferromagnetic superconductors. *Soviet Physics–JETP* **4**, 153 (1956).

45. Zh. Devizorova, S. Mironov, A. Buzdin, Theory of magnetic domain phases in ferromagnetic superconductors. *Phys. Rev. Lett.* **122**, 117002 (2019).

46. M. Kano, Y. Kohama, D. Graf, F. Balakirev, A. S. Sefat, M. A. Mcguire, B. C. Sales, D. Mandrus, S. W. Tozer, Anisotropy of the upper critical field in a co-doped $BaFe_2As_2$ single crystal. *J. Physical Soc. Japan* **78**, 084719 (2009).

47. N. Ni, M. E. Tillman, J.-Q. Yan, A. Kracher, S. T. Hannahs, S. L. Bud'ko, P. C. Canfield, Effects of Co substitution on thermodynamic and transport properties and anisotropic H_{c2} in $Ba(Fe_{1-x}Co_x)_2As_2$ single crystals. *Phys. Rev. B* **78**, 214515 (2008).

48. M. Hemmida, N. Winterhalter-Stocker, D. Ehlers, H.-A. K. von Nidda, M. Yao, J. Bannies, E. D. L. Rienks, R. Kurleto, C. Felser, B. Büchner, J. Fink, S. Gorol, T. Förster, S. Arsenijevic, V. Fritsch, P. Gegenwart, Topological magnetic order and superconductivity in $EuRbFe_4As_4$. *Phys. Rev. B* **103**, 195112 (2021).

49. X. Xi, Z. Wang, W. Zhao, J.-H. Park, K. T. Law, H. Berger, L. Forró, J. Shan, K. F. Mak, Ising pairing in superconducting $NbSe_2$ atomic layers. *Nat. Phys.* **12**, 139–143 (2016).

50. J. M. Lu, O. Zheliuk, I. Leermakers, N. F. Q. Yuan, U. Zeitler, K. T. Law, J. T. Ye, Evidence for two-dimensional Ising superconductivity in gated MoS_2. *Science* **350**, 1353–1357 (2015).

51. W. Fang, K. D. Belashchenko, M. Haim, M. Khodas, I. I. Mazin, Interplay of magnetic field and magnetic impurities in Ising superconductors. arXiv: 2306.01700 [cond-mat.supr-con] (2 June 2023).

52. E. G. Arnault, A. H. Al-Tawhid, S. Salmani-Rezaie, D. A. Muller, D. P. Kumah, M. S. Bahramy, G. Finkelstein, K. Ahadi, Anisotropic superconductivity at $KTaO_3$(111) interfaces. *Sci. Adv.* **9**, eadf1414 (2023).

53. D. Jiang, T. Yuan, Y. Wu, X. Wei, G. Mu, Z. An, W. Li, Strong In-plane magnetic field-induced reemergent superconductivity in the van der waals heterointerface of $NbSe_2$ and $CrCl_3$. *ACS Appl. Mater. Interfaces* **12**, 49252–49257 (2020).

54. H. Zhao, R. Blackwell, M. Thinel, T. Handa, S. Ishida, X. Zhu, A. Iyo, H. Eisaki, A. N. Pasupathy, K. Fujita, Smectic pair-density-wave order in $EuRbFe_4As_4$. *Nature* **618**, 940–945 (2023).

55. A. A. Bukharaev, A. K. Zvezdin, A. P. Pyatakov, Y. K. Fetisov, Straintronics: A new trend in micro- and nanoelectronics and materials science. *Phys. Usp.* **61**, 1175–1212 (2018).

56. R. Prozorov, M. A. Tanatar, N. Ni, A. Kreyssig, S. Nandi, S. L. Bud'ko, A. I. Goldman, P. C. Canfield, Intrinsic pinning on structural domains in underdoped single crystals of $Ba(Fe_{1-x}Co_x)_2As_2$. *Phys. Rev. B* **80**, 174517 (2009).

57. G. Wang, W. R. Meier, W. E. Straszheim, J. Slagle, S. L. Bud'ko, P. C. Canfield, Lack of superconductivity in the phase diagram of single-crystalline $Eu(Fe_{1-x}Co_x)_2As_2$ grown by transition metal arsenide flux. *Phys. Rev. Mater.* **2**, 104801 (2018).

-
